# Studying Structural Details in Complex Samples: II. High Field Asymmetric Waveform Ion Mobility Spectrometry (FAIMS) Coupled to High Resolution Tandem Mass Spectrometry (MS/MS)

**DOI:** 10.1021/jasms.4c00227

**Published:** 2024-11-25

**Authors:** Alessandro Vetere, Wolfgang Schrader

**Affiliations:** Max-Planck-Institut für Kohlenforschung, Kaiser-Wilhelm-Platz 1, D-45470 Mülheim (Ruhr), Germany

## Abstract

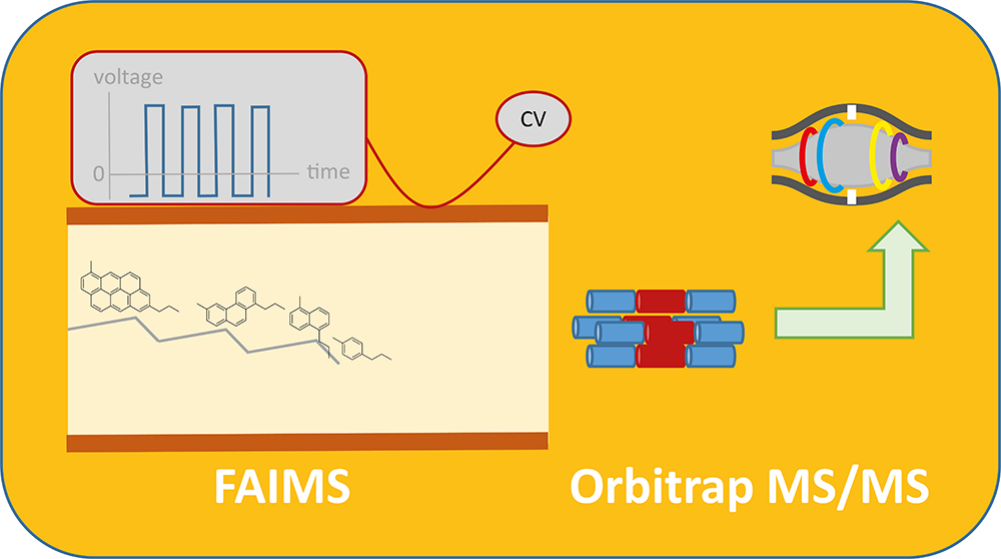

The elucidation of structural motifs in extremely complex mixtures is very difficult since the standard methods for structural elucidation are not capable to provide significant information on a single molecule. The best method for the analysis of complex mixtures is ultrahigh resolution mass spectrometry, but the utilization of this method alone does not provide significant information about structural details. Here, a combination with a separation method is necessary. While chromatography is a well-established technique, it has some disadvantages in regard to the separation of complex mixtures, as often no separation of individual isomers is possible. Therefore, here the combination of an ion mobility separation with ultrahigh resolution mass spectrometry is evaluated. As a sample matrix, crude oil is used because it is an excellent matrix to develop new analytical techniques on complex samples. Crude oil is the most complex natural sample known, but only little information is available on the structural identity or functionalities due to a high number of structural isomers or isobars. A lab-built APPI/APLI-FAIMS source was revised to optimize ion transmission and used to follow up on the ion mobility of crude oil constituents after photoionization. An MS/MS approach using collision-induced dissociation (CID) was used to elucidate structural motifs of the transmitted isomers.

## Introduction

During the past few years, the capabilities of analytical methods are getting way more powerful, allowing scientists to analyze problems with more sophisticated instrumentation than ever before. This opens up the door to new and far ranging problems that a scientist would not have thought of before. Modern analytical methods allow covering much more complex problems, including applications in areas such as synthesis, where one-pot multicomponent reactions like cascade reactions are becoming state-of-the-art.^1−3^ In environmental analysis, more complex systems are being analyzed,^4−7^ and in energy related areas, biofuels and fossil fuels from different origins are being analyzed with better resolution gaining better information on the molecular level than ever before.^8−14^

A prime example for complex analytical problems is the analysis of crude oil with its high complexity where more than one million different chemical compounds are expected to be present.^15^ Petroleum was analyzed by mass spectrometry even before this technique was widely recognized.^16^ Over the past decades, a variety of analytical techniques have been developed for, or adapted to the analysis of crude oil, many of which focusing on certain bulk parameters like boiling point distribution, density, overall aromaticity or heteroatom content.^17−23^ While all of these parameters are crucial for the refining process, information on the molecular level becomes increasingly important.

Substantial progress in this field has been made with the introduction of ultrahigh resolving mass spectrometry together with soft, nonfragmenting, ionization methods.^9,11,24−31^ Fourier transform ion cyclotron resonance mass spectrometry (FT-ICR MS) and FT-Orbitrap MS (together termed as FTMS) yield subppm mass accuracy and a mass resolving power *R* > 10^5^ (fwhm), thus allowing the unambiguous determination of the elemental composition that corresponds to a mass spectrometric signal. While numerous studies have shown that every preseparation step is beneficial for the analysis, as it reduces the ever present problem of discriminating effects,^27,32,33^ it is thus in principle possible to tackle most constituents within a crude oil by ultrahigh resolving mass spectrometry alone. Still, one major problem of the mass spectrometric approach is the inability to distinguish the broad variety of isomeric species present within a single crude oil sample without prior separation.

A chromatographic preseparation of isomeric compounds before a mass spectrometric analysis is typically done by one- or two-dimensional (GC×)GC-MS.^4,34−36^ With these techniques, structural isomers, at least with a low degree of alkylation, and especially functional isomers can be separated from each other easily. However, a gas chromatographic analysis is only suitable for relatively light oils or fractions such as kerosene. Other separation methods, as different types of liquid chromatography, e.g. RP-HPLC, ligand exchange chromatography or size-exclusion chromatography have been employed over the years, but show diverse results, when it comes to the differentiation of isomeric compounds.^37−41^ In a parallel study, we have used a preseparation with 2-fold liquid chromatography, utilizing both size-exclusion chromatography and argentation chromatography as separation steps before using ultrahigh resolution MS with collision-induced dissociation for structural analysis.^42^ The main focus in that study was the general reduction of sample complexity at any given time during the final analysis. Due to the nature of the employed chromatographic techniques, however, a distinction of isomeric species only plays a minor role. One additional problem of multidimensional liquid chromatography is that the different types of mixtures have to be soluble in the mobile phase of every dimension, which is far more difficult for complex samples such as a heavy crude oil.

For heavier oils or fractions thereof ion mobility spectrometry (IMS) is a suitable alternative that has the potential to separate isomeric compounds (ions) by their different shapes and sizes in the gas phase, hence, without an additional mobile phase.^43^ So far, most studies use IMS together with time-of-flight mass spectrometry (TOF-MS), while focusing on electrospray ionization (ESI).^44−47^ Le Maître and co-workers successfully used ion mobility on a ToF-MS together with FT-ICR-MS/MS to elucidate structural motifs of nitrogen species in crude oil related materials.^48,49^ While Szykuła and co-workers used FAIMS in a hyphenation with HESI-FTMS to increase the amount of detectable analytes in a crude oil mixture, they did not use the mobility device for isobar/isomer separation.^50^

In order to overcome the limitation of analyzing only the polar compounds in a crude oil, we previously introduced a new source block design that allows to use photoionization for FAIMS-FTMS.^51^ The setup has been revised for better ion transmission and the new design was used for MS/MS studies after separation by FAIMS. These experiments should also allow a deeper insight into structural motifs that are present in typical crude oil constituents. To this extent MS/MS has been used before, albeit often without a preseparation step that is known for its capability of isomer separation.^52−54^

## Experimental Section

### Sample Preparation

A North American heavy crude oil was diluted in toluene to a final concentration of 500 μg mL^–1^ and then analyzed without further treatment.

### Instruments and Methods

Mass spectra were recorded on a research-type Orbitrap Elite mass spectrometer^25^ (Thermo Scientific, Bremen, Germany) equipped with a FAIMS unit (Thermo Scientific, San Jose, CA, USA), while injecting the sample at a flow rate of 20 μL min^–1^. For use of photoionization (APPI) a previously described^51^ laboratory-built source block was optimized as described below. Ionization was performed by a Kr VUV lamp at 10.0 and 10.6 eV for APPI (Syagen Technologies, Tustin, CA, USA).

Positive mode mass spectra were recorded in selected ion monitoring (SIM) mode using 30 Da mass windows for monitoring the IMS behavior of preselected ions, while scanning the compensation voltage (CV) from −39 V to −9 V in steps of 0.2 V. The dispersion voltage was held at −5.0 kV with a carrier gas flow of 4.0 L min^–1^ (N_2_:He, 1:1). For each CV step, a SIM spectrum of ±15 Da around a preselected *m*/*z* (FTMS) was recorded, followed by an additional MS/MS scan after isolation and fragmentation (collision energy of 35 eV) of a preselected ion by collision-induced dissociation (CID) in the linear ion trap (LTQ).

Fragment spectra were recorded using FTMS in full scan mode. In all cases a total of 6 microscans was summed at a mass resolving power of 480,000 (fwhm at *m*/*z* 400).

## Results and Discussion

### Improvements Made to the FAIMS System

**Figure 1:**
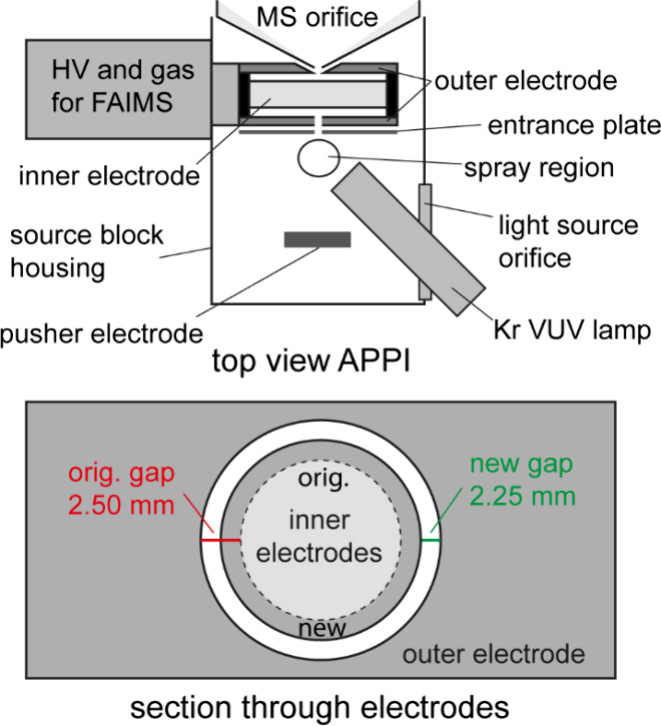
Schematic section of the reviewed source block design for APPI-FAIMS-FTMS. Top: The pusher electrode is located behind the spray region such that formed ions are accelerated into the FAIMS unit. Not shown is the thermal sprayer located at the top of the view. Bottom: Section through the electrode setup, showing the original (factory) and reduced analytical gap.

The initial FAIMS-FTMS system suffered from one major problem. The commercial Thermo FAIMS unit uses a bias voltage of 1 kV on the entrance plate. While this is not an issue when using electrospray ionization, it is a problem with ion sources that do not need a high voltage potential for ionization like APPI. To prevent ions being repelled from the FAIMS unit entrance and thus improve the sensitivity, an additional pusher electrode was installed as shown in Figure 1 (top). The electrode was operated externally by a high voltage power supply (PNC 30000-2, Heinzinger electronic GmbH, Rosenheim, Germany). After optimization on selected reference compounds (see also Supporting Information, Figure S1), the pusher was held at a voltage of 1.5 kV.

As Barnett and co-workers have previously shown, the resolution of a cylindrical FAIMS unit, as used here, can be improved by narrowing the analytical (electrode) gap.^55^ This approach was followed here. Therefore, additionally to the pusher electrode, the setup of the FAIMS unit was modified by replacing the inner electrode with a lab-built one that reduces the electrode gap from the standard of 2.50 mm to 2.25 mm (see Figure 1, bottom).

**Figure 2:**
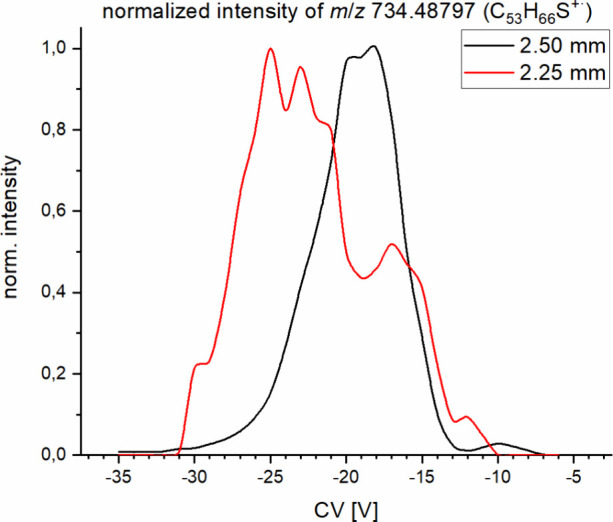
Normalized signal intensity of *m*/*z* 734.488 (corresponding to a radical ion of composition C_53_H_66_S) throughout a FAIMS separation using the standard electrodes (black line) and the modified electrodes with smaller gap (red line).

The effect of the narrower gap can be seen in Figure 2 using the transmission of ions at *m*/*z* 734.488 as an example. These traces show the transmission behavior of ions that correspond to an elemental composition of C_53_H_66_S. While with the standard electrode setup (2.50 mm gap) one peak with a transmission window between compensation voltages of −30 V and −13 V appears, the new setup displays a much better separation efficiency. When using the reduced electrode gap, the mass trace becomes much more structured, showing a multimodal distribution. By the resemblance of this signal, it is indicated that at least two to three different structural types of isomers are now partly separated that were transmitted concurrently by the standard electrode set. Also for the ions studied here, the mass trace becomes more structured, indicating a generally better separation (see Figure S2 and S3). Hence, with the new setup, isomeric compounds in complex mixtures can be at least partly separated from each other, thus allowing a more detailed structural analysis. However, high amounts of overlap still exist, and a clean isolation of single isomers is still not possible. This can also be seen in Figure S2 and S3 where MS^2^ spectra corresponding to local maxima from parent ion transmission are compared. There are overall large similarities in the fragment spectra, with differences being mostly in details of intensity distributions. Given the expected peak capacity of 10–30 (considering a width of around 5–10 V, as shown in Figure 2, per compound in FAIMS separation and a scan range of ∼30 V this makes up this peak capacity) and the expected number of different isomers higher than 10^3^, this is to be expected. In all cases studied here, the compensation voltage needed to transmit the ion is shifted by about 5–10 V toward higher absolute values (more negative in this case), as was already reported by Barnett and Oullette.^55^ The shift in compensation voltage can be attributed to the increased electric field between the electrodes as compared to the original setup.

### Effect of FAIMS toward Isolation Efficiency

**Figure 3:**
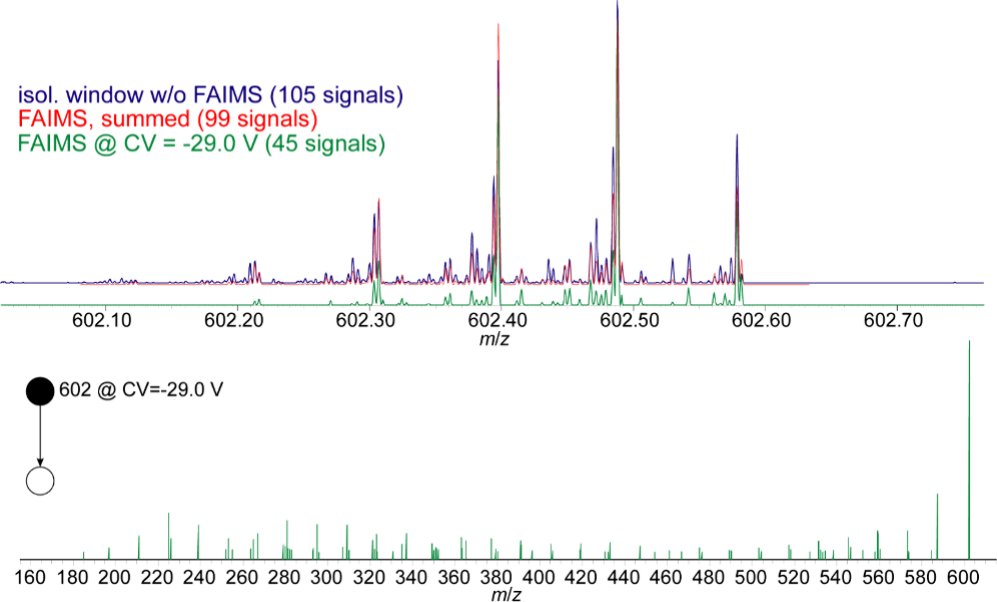
Isolation window around *m*/*z* 602 once as direct infusion (top, blue line) and once with the FAIMS unit (summed over all compensation voltages, top, red line) and once with the FAIMS unit at a CV of −29.0 V (top, green line). The bottom trace shows the resulting fragment spectrum at a CV of −29 V (MS/MS scan).

For this study the IMS transmission of selected heteroatomic compounds was monitored and their fragmentation behavior recorded after CID. Figure 3 shows the isolation window around a signal at *m*/*z* 602 with and without the FAIMS unit as well as the resulting MS/MS scan after FAIMS filtering.

The isolation device here is a linear ion trap, which does not allow very small isolation windows. Therefore, it is not possible to obtain a completely pure parent ion signal when isolating a given mass within such a complex sample. As a result, multiple isobaric compounds are still present within the isolation window that belong to different elemental compositions. In our parallel study, the amount of signals in the isolation window was drastically reduced by employing a multidimensional chromatographic separation.^42^ Here, the amount of signals in the isolation window has been reduced using an ion mobility separation. For example, the isolation window around *m*/*z* 602 contained 105 signals, when performing a direct infusion without IMS separation, the largest of which corresponds to a radical ion of C_42_H_66_S^•+^, as discussed below. When employing the FAIMS unit, this amount is only reduced by a small amount, when summing over the entire CV range (99 signals are still found). This shows that there is no significant reduction in number of signals, when using the FAIMS unit. At a given compensation voltage, however, the amount of signals in the isolation window is efficiently reduced, e.g. to only 45 signals at a compensation voltage of −29 V (see Table S1 for a detailed assignment of signals with and without using the FAIMS and Table S3 for a similar comparison regarding the nitrogen-containing compound at *m*/*z* 266 discussed below). Thus, allowing a more comprehensive analysis of the desired analytes. Compared to the direct infusion, relative signal intensities are shifted throughout the IMS separation, as can for example be seen with the signal group around *m*/*z* 602.47 in Figure 3. This can be attributed to different mobilities of different isomers that vary in abundance. Therefore, different amounts of isobaric species are present in the isolation window at a given point in time (corresponding to a compensation voltage).

Although the isolation window is significantly less crowded, when using FAIMS, fragments generated in the MS/MS scan still originate from various other compounds, as can be seen from Table S2 (see also Table S4 for a similar comparison regarding the nitrogen-containing compound at *m*/*z* 266 discussed below). Most of these can be easily identified as belonging to coisolated isobars as they contain a too high number of different heteroatoms (yellow lines in Table S2) or correspond to an unrealistically high DBE (red lines in Table S2).

### Structural Elucidation of Individual Compounds in Complex Mixtures by FAIMS-MS/MS

#### Example 1: Radical Cation C_42_H_66_S^•+^

The optimized experimental setup was used to gain structural insights about functionalities of individual compounds present in a very heavy crude oil. Radical cations detected at *m*/*z* 602.48797 (the most abundant signal in Figure 3) have an elemental composition of C_42_H_66_S, corresponding to a double bond equivalent (DBE) of 10.

**Figure 4:**
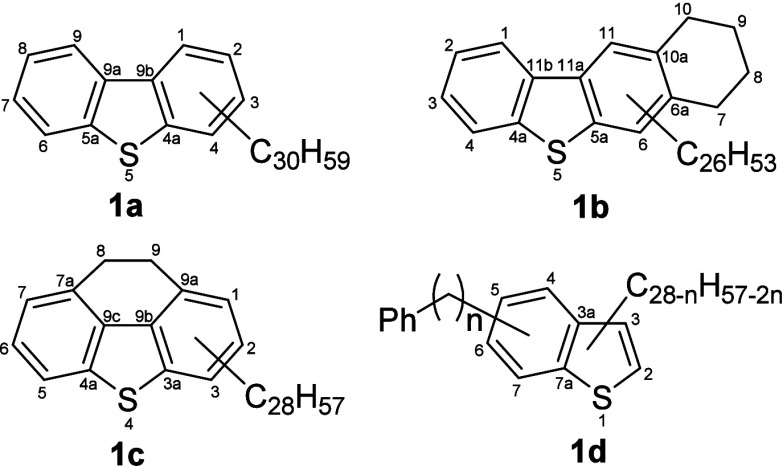
Possible isomeric structure types for C_42_H_66_S. Dibenzothiophenic (**1a**), tetrahydrobenzonaphthothiophenic (**1b**), dihydrophenanthrothiophenic (**1c**) or phenylated benzothiophenic (**1d**) structures are possible. Indicated alkyl chains might be split up into smaller substituents. Atom positions on the aromatic core are indicated as numbers.

Such sulfur-containing compounds are considered to be mostly thiophenic. Some possible structure types are shown in Figure 4. The possibilities include structures derived from benzo- (**1d**), dibenzo- (**1a**), benzonaphtho- (**1b**) or phenanthrothiophenes **(1c**). Generally, sulfidic compounds or mercaptans would also be possible, but are not considered to be an equally important group of compounds in a heavy crude oil.

**Scheme 1:**
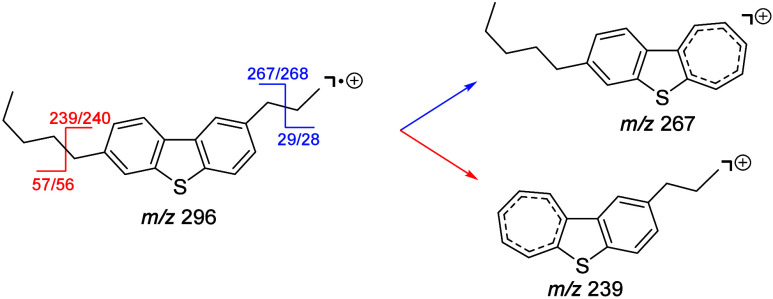
Basic Mechanism for the Fragmentation of Polycyclic Aromatic Compounds Dominant is the homolytic cleavage in a benzylic position. With alkyl chains of three or more carbon atoms the fragmentation reaction competes with a McLafferty rearrangement that results in the loss of an alkene.^57^

**Figure 5:**
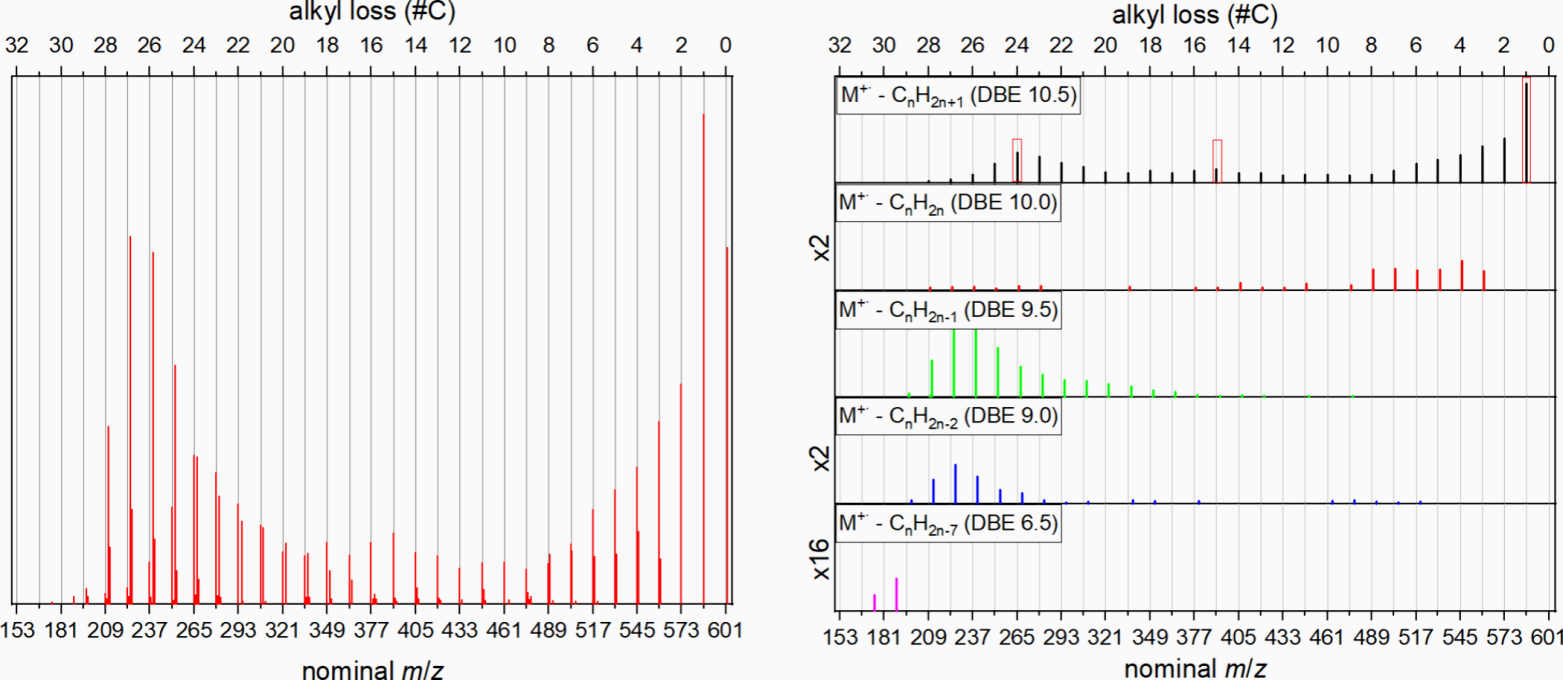
Fragment ion spectra of *m*/*z* 602.49 (C_42_H_66_S^•+^) after summation over the entire CV range. The bottom axes show the nominal *m*/*z* of detected fragment ions, while the top axes show the number of carbon atoms lost during fragmentation. Left panel: Whole spectrum. Right panel: Spectrum separated into different series of fragment ions, corresponding to the indicated DBE values, where applicable zoom factors are indicated on the left side. Shown fragment ions represent those fragments that are considered for interpretation only (compare Table S2). Overall, 99 of 528 signals in the summarized spectrum were used for interpretation for the suggested structures.

The cumulative fragment ion spectra obtained from these precursor ions while scanning the CV of the FAIMS unit are shown in Figure 5 on the left side. For a better overview, the top axis shows the number of carbon atoms lost to generate the corresponding fragment. On first sight, the observed 14 Da pattern does resemble electron impact (EI) spectra of alkyl chains with cleavage at arbitrary positions, as was also suggested by Porter and co-workers.^56^ However, we have recently shown that crude oil relevant, aromatic compounds fragment predominantly by benzylic cleavage leading to a single fragment signal of each alkyl chain.^57^ This is exemplarily shown in Scheme 1 for a disubstituted dibenzothiophene.

The DBE 10.5 fragment series (Figure 5), however, shows local maxima at 1, 15, and 24 carbon atoms to be lost, indicating the presence of high amounts of ethyl, hexadecyl and pentaeicosyl side chains. The highest possible loss of a single alkyl chain by benzylic cleavage for type **1b** compounds is of C_26_H_53_ (if the chain is located in position 7 or 10, C_25_H_51_ otherwise); for type **1c** compounds, the highest possible loss is of C_28_H_57_ (if chain is located in position 8/9, C_27_H_55_ otherwise); and for type **1d** compounds, it is of C_27_H_55_ (if n = 0). Given that for type **1a** compounds the alkyl chains need to contain one additional double bond or ring, the maximum alkyl cleavage would be of C_27_H_55_ for an octaeicosyl chain and an additional ethylene substituent. The smallest fragment observed within the DBE 10.5 series is at *m*/*z* 209, corresponding to a loss of C_28_H_57_. This is therefore indicative of a compound of type **1c** with a single alkyl chain located in position 8 (or 9 which are equal).

**Figure 6:**
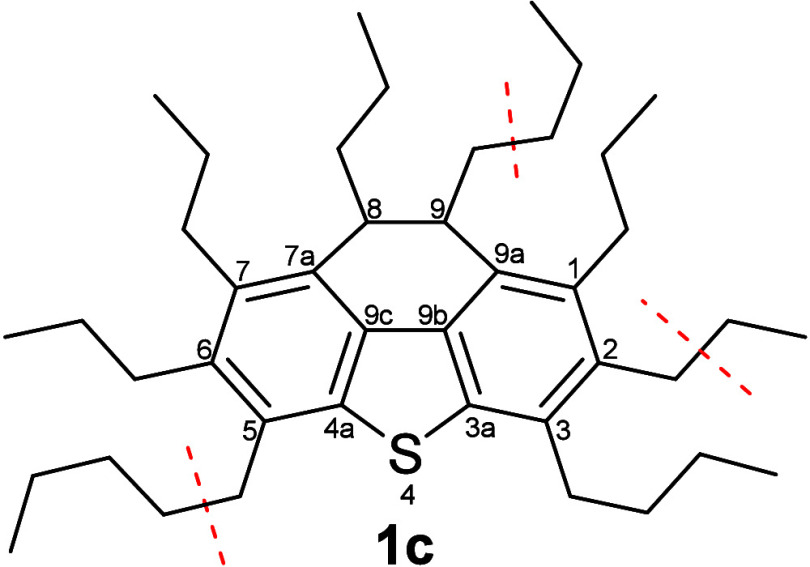
Example of a phenanthrothiophenic **1c**-type structure with assumed statistical distribution of alkyl chains around the aromatic core. The resulting alkyl chains have a length of 3–5 carbon atoms. Examples of benzylic cleavages of ethyl, propyl or butyl fragments are indicated by dashed lines.

For these compounds, it can be concluded that a statistical distribution of alkyl chains, as exemplarily shown in Figure 6, is unlikely. Instead, the presence of only around two alkyl chains seems to be favored, where both are either of similar length (local distribution maximum around a C_15_-loss) or one is very long with the other one being very short (local maxima at losses of C_1_ and C_24_).

The fragment ion series with DBE 10.0 generally follows the same trend. This is expected, since for longer alkyl chains the rearrangement reaction^57^ is competing with the direct homolytic fission. Additionally, this series can contain fragment ions that result from retro-Diels–Alder reactions of **1b** type precursor ions. Both types of reaction cannot be distinguished here; however, the overall similarity of the intensity distribution to the DBE 10.5 series and the low intensity of the DBE 10.0 series indicate that the corresponding structure type is of relatively low abundance. The fact that the intensity observed for the DBE 10.0 series is relatively low also for the loss of long alkyl chains might be indicative for the McLafferty rearrangement being often hindered. This could be a result of neighboring ortho-positions being substituted as well.

The following fragment ion series (DBE 9.5 and 9.0) originate from the same type of fragmentation reactions but with a double bond equivalent—most probably a naphthenic ring—residing within the lost alkyl chain. These series of fragment ions therefore can be attributed to a **1a** type parent structure, where the naphthenic ring is not fused to the aromatic core (as in **1b** or **1c**). While the maximum intensity is observed for fragments after loss of a C_27_-chain, loss of small fragments is absent for these series. The lowest mass fragments at *m*/*z* 197/198, indicative of a C_29_-loss (i.e., one alkyl chain is present) are in good accordance to the proposed type **1b** structure. No information, however, is available on the exact position of any side chain, nor on its degree of branching. In the case of type **1a** parent structures, the position of the naphthenic ring inside the substituent is also not known.

**Scheme 2:**
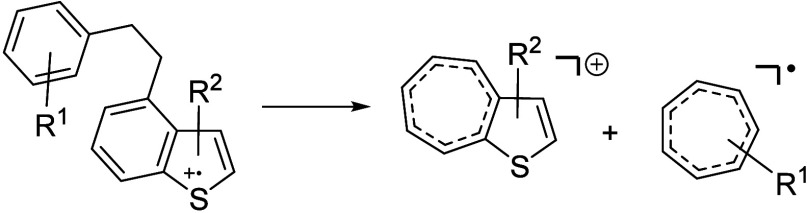
Fragmentation of a **1d** Type Compound with an Ethylene Bridge Substituent positions are examples.

Cleavage of small alkyl substituents (resulting in DBE 10.5 and 10 series fragments, see Figure 5) can occur for all four structure types proposed and are therefore not conclusive regarding the different structural possibilities. However, two low intensity indications are also found for type **1d** parent compounds. Fragment ions at *m*/*z* 175 and 189 (DBE 6.5) result from the loss of a DBE 4 fragment, which is indicative of a standalone phenyl ring. In total 31/30 carbon atoms are lost to produce these fragments, which is close to the maximum number of 33 (a total of 34 carbon atoms in side chains). The absence of higher mass fragments within this series reveals that the phenyl substituent, while not fused to the aromatic benzothiophenic core, must still be in close vicinity to it, probably separated by a small alkyl bridge of only 2 to 4 carbon atoms (compare Scheme 2). When further away from the aromatic core, other fragments of higher mass that result from the benzylic cleavage next to the phenyl moiety should be present. Additionally this phenyl ring must bear the majority of the remaining, aliphatic side chains (R^1^ in Scheme 2).

Apart from the structure types discussed so far, a sulfidic compound with the sulfur residing inside an open chain or a naphthenic ring is possible. Regarding the absence of sulfur-containing low DBE fragments, such structures, however, do not seem to be very abundant.

Overall, the data show evidence for the presence of compounds of type **1d** (DBE 6.5 series of fragments), type **1a** (DBE 9.5 and 9.0 series of fragments) and type **1c** (especially fragment at *m*/*z* 209). The presence of type **1b** compounds can neither be confirmed nor ruled out, as possible fragments by retro-Diels–Alder reaction of such species and fragmentation by McLafferty rearrangement from other structural types are not distinguishable.

While it is generally a good technique for the separation of isomers, ion mobility, especially cylindrical FAIMS, is known for limited separation capabilities. Fragment ion traces from this very complex mixture do overlap within a broad CV. With the dominance of rather long alkyl chains observed, this can, however, be expected. The ion mobility of the distinct isomers, i.e. the degree of conformational change between the high and low electric field portion of the wave, will be mostly affected by the folding abilities of these substituents. Minor changes in branching or alkyl chain position will, under these circumstances, only be of limited effect. However, one major advantage of using FAIMS is that due to the intrinsic simplification of the mixture that passes the mobility unit at a given setting, discrimination effects are reduced, thus enabling the detection also of low abundant species, such as the **1d** type compounds.

#### Example 2: Protonated Molecule C_19_H_24_N^+^

**Figure 7:**
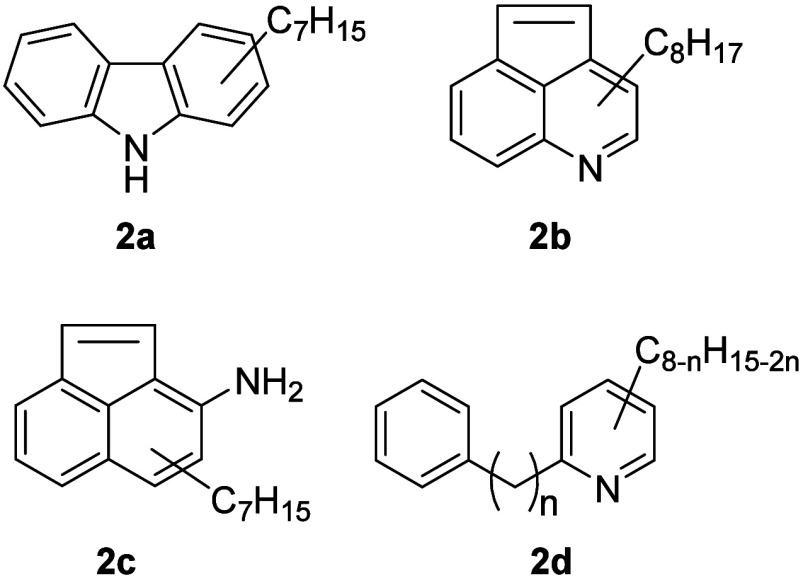
Possible isomeric structure types for C_19_H_23_N. Indicated alkyl chains might be split up into several smaller substituents, including possible *N*-alkylation for types **2a** and **2c**.

Cations detected at *m*/*z* 266.19033 result from parent compounds of composition C_19_H_23_N that have been ionized by protonation. Corresponding molecules bear a DBE of 9, with the observed ions being of DBE 8.5. While such species are mostly considered as being carbazole type compounds (see Figure 7, **2a**), also pyridinic (**2b** and **2d**) or aniline related structure types (**2c**) are possible.

**Figure 8:**
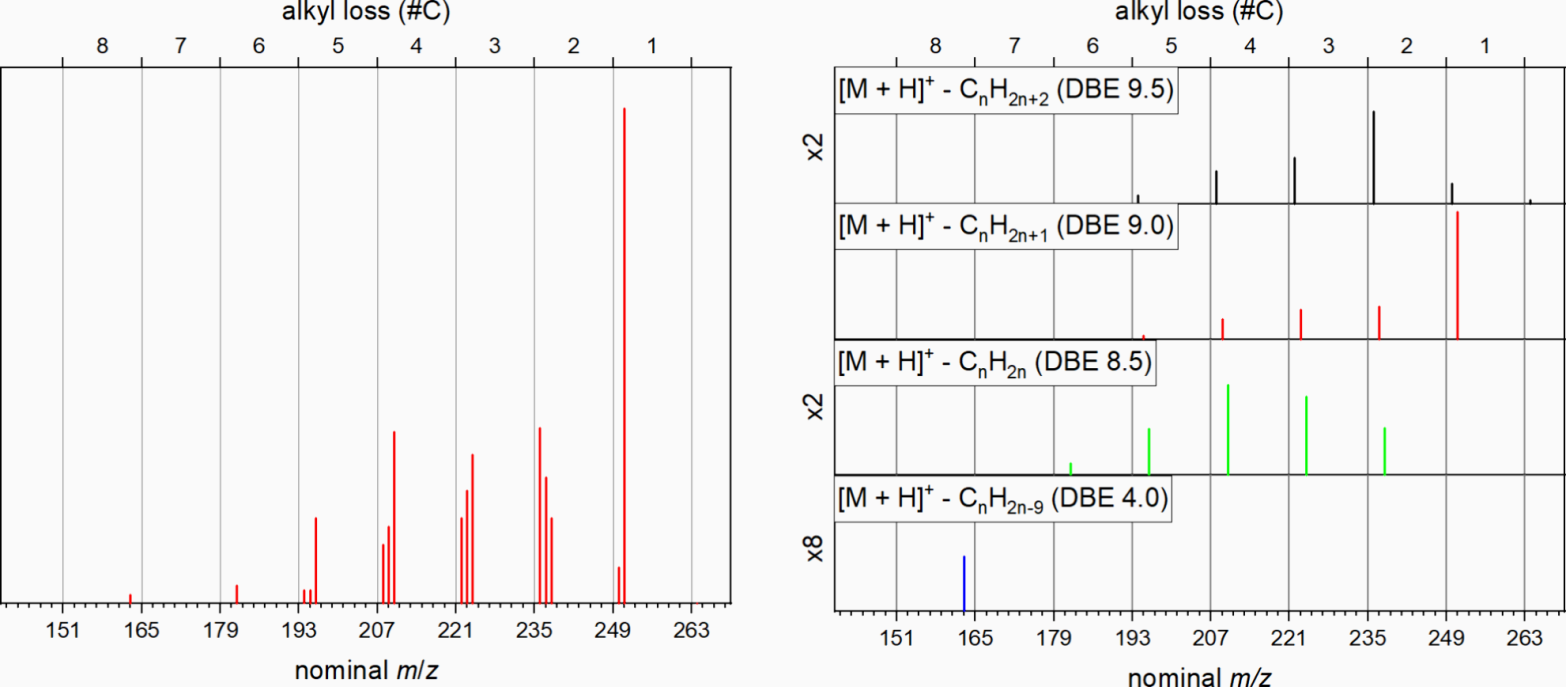
Fragment ion spectra of *m*/*z* 266.19 ([C_19_H_23_N+H]^+^_)_ after summation over the entire CV range. The bottom axes show the nominal *m*/*z* of detected fragments ions, while the top axes show the number of carbon atoms lost during fragmentation. Left panel: Summarized spectrum throughout CV range. Right panel: Spectrum separated into different series of fragment ions, corresponding to the indicated DBE values, where applicable zoom factors are indicated on the left side. Shown fragment ions represent those fragments that are considered for interpretation only (compare Table S4). Overall, 16 of 319 signals in the summarized spectrum were used for interpretation for the suggested structures.

Compared to the previous example the isomeric compounds investigated here are less extensively alkylated, with only 7 to 8 carbon atoms in aliphatic chains. The summarized fragment spectra shown in Figure 8 are thus much less complicated. For a comparison of isolation windows with and without FAIMS and an assessment of fragment ions, see Tables S3 and S4. Loss of a methyl group is, again, by far the dominating fragmentation. This is indicative of small alkyl chains being favored. The highest alkyl loss (Figure 8, right panel, DBE series 9.0 and 8.5) is of C_5_H_11_ and C_6_H_12_, respectively. While this is not a clear criterion against type **2b** or **2d** isomers, with a maximum alkyl loss of C_7_H_15_, this corresponds well with type **2a** or **2c** structures.

Remarkable is the relatively high abundance of DBE 9.5 fragments. These correspond to a loss of fully saturated alkanes, starting from methane, up to pentane, with the maximum intensity observed for the loss of ethane. Such behavior has so far only been reported for alkylated amines, where nitrogen is not part of an aromatic system, This also includes anilinic **2c** type compounds.^58,59^

**Scheme 3:**
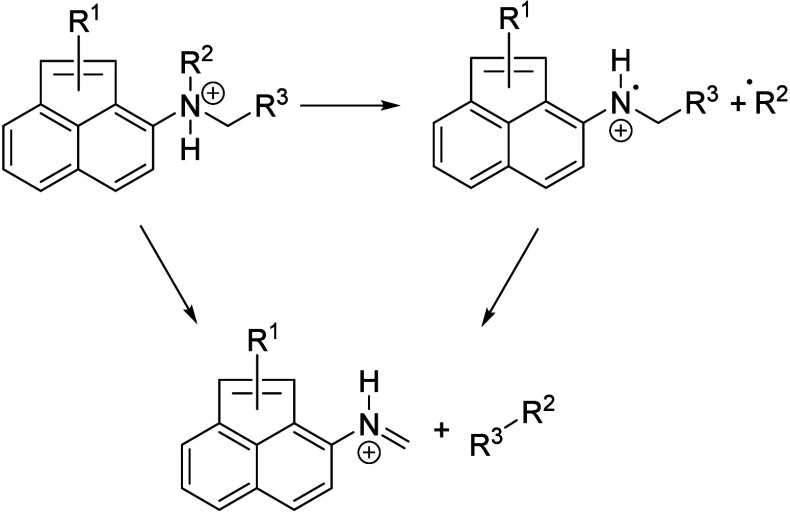
Reaction Pathways Leading to Loss of an Alkane from Alkylated Amines Either a two-step radical process or a concerted rearrangement are possible.

Two reaction pathways are discussed for this kind of fragmentation. The reaction can progress via a concerted rearrangement that leads to the removal of an alkane. Alternatively, an alkyl radical is lost from the nitrogen atom, followed by further loss of a second radical from the metastable product. Both pathways, as depicted in Scheme 3, lead to the formation of a C–N double bond and the net loss of an alkane. In case of a radical mechanism, the formation of an alkane from both radical fragments is, however, unlikely. The present data do not allow any differentiation between both pathways.

Interestingly, the loss of ethane is the second most abundant fragmentation observed for these isomeric compounds. This finding is contrasting earlier studies which report aliphatic amines or even anilinic species to be either fully absent or of relatively low abundance in a crude oil.^60−62^

Similar to the previous examples of sulfur-containing compounds, only one low intensity signal is observed that indicates the loss of an aromatic substructure. The fragment ion observed at *m*/*z* 163 (bottom trace in Figure 8, right side) results from the loss of a C_8_H_7_ radical, leaving a protonated DBE 4 fragment behind. Considering the limited possibilities for a reasonable substructure, we propose that this fragmentation originates from some kind of **2d** type pyridinic species with a styrene type substituent.

**Figure 9:**
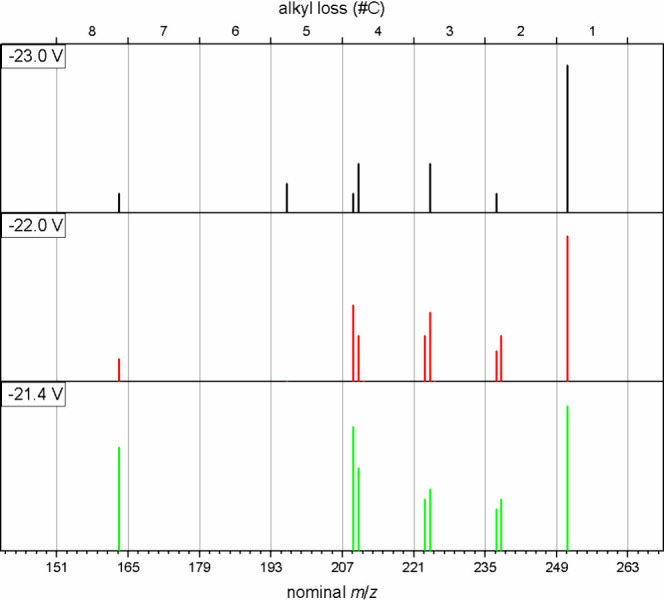
Fragment ion spectra, resulting from CID on the parent ion at *m*/*z* 266.19033. Spectra were recorded at compensation voltages, leading to a local maximum for the *m*/*z* 163 fragment.

For this fragment, three local maxima are observed at a CV of −23.0 V, −22.0 V and −21.4 V. The fragment spectra obtained at these compensation voltages are shown in Figure 9. As a full separation of all isomeric compounds cannot be assumed, however, not all observed signals will originate from a corresponding **2d** type structure.

**Scheme 4:**
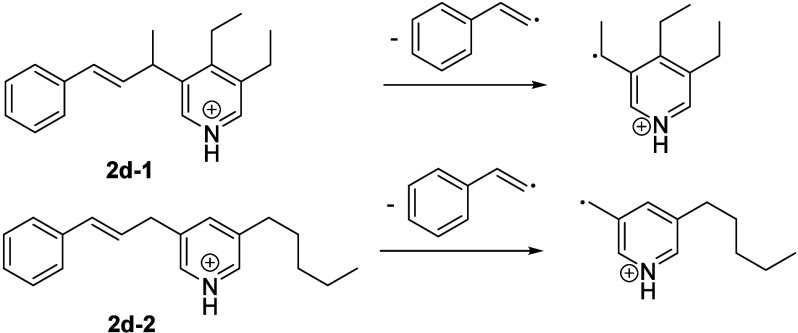
Proposed **2d** Type Parent Structures That Might Lead to the Fragmentation Pattern Shown in Figure 9

Differences in details can be observed, especially between the spectra recorded at −23.0 V and −21.4 V. In the first case, loss of a methyl group is the dominating fragmentation pathway, indicating the presence of multiple short alkyl chains, while styryl loss yields only about 10% of the total intensity. For the isomeric compound transmitted at −21.4 V however, losses of a styryl or a butyl radical (indicating a pentyl substituent) are almost equally abundant. Scheme 4 shows two possible isomeric structures that might lead to the fragmention pattern observed at −23 V (**2d-1**) and −21.4 V (**2d-2**). To the best of our knowledge, this type of structural motifs have not yet been reported to be present in crude oil.

Overall, the fragmentation found for the C_19_H_23_N series of compounds is not completely conclusive regarding types **2a** and **2b**. The fragments of DBE series 8.5 and 9.0 could be attributed to any of the discussed structural types. Therefore, no differentiation is possible. However, the DBE 9.5 series of fragments is a strong indication of **2c** type anilinic compounds. Pyridinics of type **2d** with a remote phenyl substituent are evidenced by the DBE 4.0 fragments.

## Conclusions

Ultrahigh resolving mass spectrometry has enabled huge progress in a complex mixture analysis by its potential to determine elemental compositions of the various analytes present. Still, assumptions on the structure and therefore compound types of detected species in this crude oil mixture mostly rely on the heteroatom content, double bond equivalent and—mostly—experience only. This will arguably lead to an oversimplification, as it limits the findings to the most common compound classes. In this study, we used FAIMS with a custom-made, optimized source block for APPI-MS/FTMS of a heavy crude oil.

With this setup we were able to obtain a much cleaner isolation window for MS/MS measurements, which in turn allowed gaining a deeper look onto the structural details and isomeric variances of selected compounds.

Thus, previously unsought structural motifs have been identified successfully.

In comparison to the multidimensional chromatographic approach, followed in part I of this study, the use of ion mobility benefits from two advantages: First, being a single-step online approach, it does not require lengthy preseparation and collection steps. Second, it mitigates potential problems of solvent incompatibilities between the chromatographic steps. However, it does not introduce a separation based on chemical properties and also not directly based on functionalities, as the separation mechanism does not rely on the collisional cross section alone, but rather on the change thereof in the low- and high-field regime of the electric wave. Therefore, it does not allow any selectivity to certain compound groups, which is the strength of chromatography.

## Supporting Information Available

Details for FAIMS pusher electrode optimization, Ion transmission curves for signals discussed in this study, isolation window around *m*/*z* 602 - peak annotation, MS/MS scan of *m*/*z* 602 at CV −29 V - peak annotation, MS/MS scan of *m*/*z* 266 at CV −26.4 V - peak annotation (PDF)

## References

[ref1] Oliveira C. C.; Marques M. V.; Godoi M. N.; Regiani T.; Santos V. G.; Santos E. A. F. d.; Eberlin M. N.; Sa M. M.; Correia C. R. D. Chemo-, Regio- and Stereoselective Heck Arylation of Allylated Malonates: Mechanistic Insights by ESI-MS and Synthetic Application toward 5-Arylmethyl-gamma-lactones. Org. Lett. 2014, 16 (19), 5180–5183. 10.1021/ol502529v. 25247735

[ref2] Santos V. G.; Godoi M. N.; Regiani T.; Gama F. H. S.; Coelho M. B.; de Souza R. O. M. A.; Eberlin M. N.; Garden S. J. The Multicomponent Hantzsch Reaction: Comprehensive Mass Spectrometry Monitoring Using Charge-Tagged Reagents. Chem. - Eur. J. 2014, 20 (40), 12808–12816. 10.1002/chem.201303065. 25179028

[ref3] Alachraf M. W.; Wende R. C.; Schuler S. M. M.; Schreiner P. R.; Schrader W. Functionality, Effectiveness, and Mechanistic Evaluation of a Multicatalyst-Promoted Reaction Sequence by Electrospray Ionization Mass Spectrometry. Chem. - Eur. J. 2015, 21 (45), 16203–16208. 10.1002/chem.201502640. 26407155

[ref4] Barrow M. P.; Peru K. M.; Headley J. V. An Added Dimension: GC Atmospheric Pressure Chemical Ionization FTICR MS and the Athabasca Oil Sands. Anal. Chem. 2014, 86 (16), 8281–8288. 10.1021/ac501710y. 25036898

[ref5] Jennings E.; Kremser A.; Han L.; Reemtsma T.; Lechtenfeld O. J. Discovery of Polar Ozonation Byproducts via Direct Injection of Effluent Organic Matter with Online LC-FT-ICR-MS. Environ. Sci. Technol. 2022, 56 (3), 1894–1904. 10.1021/acs.est.1c04310. 35007417

[ref6] Chen M.; Wei D. B.; Li L. P.; Wang F. P.; Du Y. G. Magnitude Filter Combined with Mass Filter: A Reliable Strategy to Improve the Reproducibility of ESI-FT-ICR-MS Analysis on the Fingerprint of Dissolved Organic Matter. Anal. Chem. 2022, 94 (30), 10643–10650. 10.1021/acs.analchem.2c00879. 35830694

[ref7] Luo R. J.; Schrader W. Getting a better overview of a highly PAH contaminated soil: A non-targeted approach assessing the real environmental contamination. J. Hazard. Mater. 2021, 418, 126352 10.1016/j.jhazmat.2021.126352. 34329030

[ref8] Abou-Dib A.; Aubriet F.; Hertzog J.; Vernex-Loset L.; Schramm S.; Carré V. Next Challenges for the Comprehensive Molecular Characterization of Complex Organic Mixtures in the Field of Sustainable Energy. Molecules 2022, 27 (24), 8889 10.3390/molecules27248889. 36558021 PMC9786309

[ref9] Miettinen I.; Kuittinen S.; Paasikallio V.; Mäkinen M.; Pappinen A.; Jänis J. Characterization of fast pyrolysis oil from short-rotation willow by high-resolution Fourier transform ion cyclotron resonance mass spectrometry. Fuel 2017, 207, 189–197. 10.1016/j.fuel.2017.06.053.

[ref10] Lalli P. M.; Jarvis J. M.; Marshall A. G.; Rodgers R. P. Functional Isomers in Petroleum Emulsion Interfacial Material Revealed by Ion Mobility Mass Spectrometry and Collision-Induced Dissociation. Energy Fuels 2017, 31 (1), 311–318. 10.1021/acs.energyfuels.6b02411.

[ref11] Palacio Lozano D. C.; Gavard R.; Arenas-Diaz J. P.; Thomas M. J.; Stranz D. D.; Mejía-Ospino E.; Guzman A.; Spencer S. E. F.; Rossell D.; Barrow M. P. Pushing the analytical limits: new insights into complex mixtures using mass spectra segments of constant ultrahigh resolving power. Chem. Sci. 2019, 10 (29), 6966–6978. 10.1039/C9SC02903F. 31588263 PMC6764280

[ref12] Neumann A.; Kafer U.; Groger T.; Wilharm T.; Zimmermann R.; Ruger C. P. Investigation of Aging Processes in Bitumen at the Molecular Level with High-Resolution Fourier-Transform Ion Cyclotron Mass Spectrometry and Two-Dimensional Gas Chromatography Mass Spectrometry. Energy Fuels 2020, 34 (9), 10641–10654. 10.1021/acs.energyfuels.0c01242.

[ref13] Alzarieni K. Z.; Zhang Y.; Niyonsaba E.; Wehde K. E.; Johnston C. T.; Kilaz G.; Kenttämaa H. I. Determination of the Chemical Compositions of Condensate-like Oils with Different API Gravities by Using the Distillation, Precipitation, Fractionation Mass Spectrometry (DPF MS) Method. Energy Fuels 2021, 35 (10), 8646–8656. 10.1021/acs.energyfuels.0c04286.

[ref14] Buss W.; Hertzog J.; Pietrzyk J.; Carre V.; Mackay C. L.; Aubriet F.; Masek O. Comparison of Pyrolysis Liquids from Continuous and Batch Biochar Production-Influence of Feedstock Evidenced by FTICR MS. Energies 2021, 14 (1), 9 10.3390/en14010009.

[ref15] Beens J.; Blomberg J.; Schoenmakers P. J. Proper tuning of comprehensive two-dimensional gas chromatography (GC x GC) to optimize the separation of complex oil fractions. J. High. Resolut. Chromatogr. 2000, 23 (3), 182–188. 10.1002/(SICI)1521-4168(20000301)23:3<182::AID-JHRC182>3.0.CO;2-E.

[ref16] Griffiths J. A Brief History of Mass Spectrometry. Anal. Chem. 2008, 80 (15), 5678–5683. 10.1021/ac8013065. 18671338

[ref17] Espada J. J.; Fernández S.; Velasco L.; Coto B. Evaluation of different methodologies to determine the n-paraffin distribution of petroleum fractions. Fuel 2013, 109, 470–475. 10.1016/j.fuel.2013.02.057.

[ref18] O’Donnell R. J. Measurement of Fractionation in Analytical Distillation of Crude Oil. Ind. Eng. Chem. Process Des. Dev. 1973, 12 (2), 208–211. 10.1021/i260046a014.

[ref19] Alomair O.; Jumaa M.; Alkoriem A.; Hamed M. Heavy oil viscosity and density prediction at normal and elevated temperatures. J. Pet. Explor. Prod. Technol. 2016, 6 (2), 253–263. 10.1007/s13202-015-0184-8.

[ref20] Rogel E.; Ovalles C.; Bake K. D.; Zuo J. Y.; Dumont H.; Pomerantz A. E.; Mullins O. C. Asphaltene Densities and Solubility Parameter Distributions: Impact on Asphaltene Gradients. Energy Fuels 2016, 30 (11), 9132–9140. 10.1021/acs.energyfuels.6b01794.

[ref21] Jia Z.; Xiao L.; Wang Z.; Liao G.; Zhang Y.; Liang C. Molecular dynamics and composition of crude oil by low-field nuclear magnetic resonance. Magn. Reson. Chem. 2016, 54 (8), 650–655. 10.1002/mrc.4424. 26990450

[ref22] Kovalenko E. Y.; Golushkova E. B.; Sagachenko T. A. The study of the composition of oils and structure of their components during the preliminary refining of oil feedstock with metal powders. Pet. Chem. 2016, 56 (2), 101–108. 10.1134/S0965544116010047.

[ref23] Walkner C.; Gratzer R.; Meisel T.; Bokhari S. N. H. Multi-element analysis of crude oils using ICP-QQQ-MS. Org. Geochem. 2017, 103, 22–30. 10.1016/j.orggeochem.2016.10.009.

[ref24] Santos J. M.; Santos F. M. L.; Eberlin M. N.; Wisniewski A. Advanced Aspects of Crude Oils Correlating Data of Classical Biomarkers and Mass Spectrometry Petroleomics. Energy Fuels 2017, 31 (2), 1208–1217. 10.1021/acs.energyfuels.6b02362.

[ref25] Vetere A.; Schrader W. Mass Spectrometric Coverage of Complex Mixtures: Exploring the Carbon Space of Crude Oil. ChemistrySelect 2017, 2 (3), 849–853. 10.1002/slct.201601083.

[ref26] Barrow M. P.; Peru K. M.; McMartin D. W.; Headley J. V. Effects of Extraction pH on the Fourier Transform Ion Cyclotron Resonance Mass Spectrometry Profiles of Athabasca Oil Sands Process Water. Energy Fuels 2016, 30 (5), 3615–3621. 10.1021/acs.energyfuels.5b02086.

[ref27] Cho Y.; Ahmed A.; Islam A.; Kim S. Developments in FT-ICR MS instrumentation, ionization techniques, and data interpretation methods for petroleomics. Mass Spectrom. Rev. 2015, 34 (2), 248–263. 10.1002/mas.21438. 24942384

[ref28] Gaspar A.; Schrader W. Expanding the data depth for the analysis of complex crude oil samples by Fourier transform ion cyclotron resonance mass spectrometry using the spectral stitching method. Rapid Commun. Mass Spectrom. 2012, 26 (9), 1047–1052. 10.1002/rcm.6200. 22467454

[ref29] Gaspar A.; Zellermann E.; Lababidi S.; Reece J.; Schrader W. Characterization of Saturates, Aromatics, Resins, and Asphaltenes Heavy Crude Oil Fractions by Atmospheric Pressure Laser Ionization Fourier Transform Ion Cyclotron Resonance Mass Spectrometry. Energy Fuels 2012, 26 (6), 3481–3487. 10.1021/ef3001407.

[ref30] Santos J.; Pudenzi M.; Wisniewski Jr A.; Breitkreitz M.; Eberlin M. Optimization of Atmospheric Pressure Photoionization for the Crude Oil Analysis Using Ultra-High Resolution Mass Spectrometry. J. Braz. Chem. Soc. 2018, 30 (4), 819–829. 10.21577/0103-5053.20180214.

[ref31] Hertzog J.; Carré V.; Le Brech Y.; Mackay C. L.; Dufour A.; Mašek O.; Aubriet F. Combination of electrospray ionization, atmospheric pressure photoionization and laser desorption ionization Fourier transform ion cyclotronic resonance mass spectrometry for the investigation of complex mixtures - Application to the petroleomic analysis of bio-oils. Anal. Chim. Acta 2017, 969, 26–34. 10.1016/j.aca.2017.03.022. 28411627

[ref32] Giraldo-Dávila D.; Chacón-Patiño M. L.; Orrego-Ruiz J. A.; Blanco-Tirado C.; Combariza M. Y. Improving compositional space accessibility in (+) APPI FT-ICR mass spectrometric analysis of crude oils by extrography and column chromatography fractionation. Fuel 2016, 185, 45–58. 10.1016/j.fuel.2016.07.096.

[ref33] Santos J. M.; Vetere A.; Wisniewski A.; Eberlin M. N.; Schrader W. Modified SARA Method to Unravel the Complexity of Resin Fraction(s) in Crude Oil. Energy Fuels 2020, 34 (12), 16006–16013. 10.1021/acs.energyfuels.0c02833.

[ref34] Zhang W.; Zhu S.; Pang L.; Gao X.; Zhu G.-T.; Li D. Determination of diamondoids in crude oils using gas purge microsyringe extraction with comprehensive two dimensional gas chromatography-time-of-flight mass spectrometry. J. Chromatogr. A 2016, 1478, 75–83. 10.1016/j.chroma.2016.11.055. 27914609

[ref35] Genuit W.; Chaabani H. Comprehensive two-dimensional gas chromatography-field ionization time-of-flight mass spectrometry (GCxGC-FI-TOFMS) for detailed hydrocarbon middle distillate analysis. Int. J. Mass spectrom. 2017, 413, 27 10.1016/j.ijms.2016.12.001.

[ref36] Benigni P.; DeBord J. D.; Thompson C. J.; Gardinali P.; Fernandez-Lima F. Increasing Polyaromatic Hydrocarbon (PAH) Molecular Coverage during Fossil Oil Analysis by Combining Gas Chromatography and Atmospheric-Pressure Laser Ionization Fourier Transform Ion Cyclotron Resonance Mass Spectrometry (FT-ICR MS). Energy Fuels 2016, 30 (1), 196–203. 10.1021/acs.energyfuels.5b02292. 27212790 PMC4869715

[ref37] Kim D.; Jin J. M.; Cho Y.; Kim E.-H.; Cheong H.-K.; Kim Y. H.; Kim S. Combination of ring type HPLC separation, ultrahigh-resolution mass spectrometry, and high field NMR for comprehensive characterization of crude oil compositions. Fuel 2015, 157, 48–55. 10.1016/j.fuel.2015.04.061.

[ref38] Lababidi S.; Panda S. K.; Andersson J. T.; Schrader W. Direct Coupling of Normal-Phase High-Performance Liquid Chromatography to Atmospheric Pressure Laser Ionization Fourier Transform Ion Cyclotron Resonance Mass Spectrometry for the Characterization of Crude Oil. Anal. Chem. 2013, 85 (20), 9478–9485. 10.1021/ac400670s. 24063573

[ref39] Nocun M.; Andersson J. T. Argentation chromatography for the separation of polycyclic aromatic compounds according to ring number. J. Chromatogr. A 2012, 1219, 47–53. 10.1016/j.chroma.2011.11.006. 22153206

[ref40] Panda S. K.; Alawani N. A.; Lajami A. R.; Al-Qunaysi T. A.; Muller H. Characterization of aromatic hydrocarbons and sulfur heterocycles in Saudi Arabian heavy crude oil by gel permeation chromatography and ultrahigh resolution mass spectrometry. Fuel 2019, 235, 1420–1426. 10.1016/j.fuel.2018.07.118.

[ref41] Sim A.; Cho Y.; Kim D.; Witt M.; Birdwell J. E.; Kim B. J.; Kim S. Molecular-level characterization of crude oil compounds combining reversed-phase high-performance liquid chromatography with off-line high-resolution mass spectrometry. Fuel 2015, 140, 717–723. 10.1016/j.fuel.2014.10.019.

[ref42] Dreschmann J.; Guricza L. M.; Schrader W. Studying Structural Details in Complex Samples: I. Two-Dimensional Chromatography Coupled to Ultrahigh Resolution Mass Spectrometry. J. Am. Soc. Mass. Spectrom. 2024, 10.1021/jasms.4c00226. PMC11622240 39555888

[ref43] Benigni P.; Sandoval K.; Thompson C. J.; Ridgeway M. E.; Park M. A.; Gardinali P.; Fernandez-Lima F. Analysis of Photoirradiated Water Accommodated Fractions of Crude Oils Using Tandem TIMS and FT-ICR MS. Environ. Sci. Technol. 2017, 51 (11), 5978–5988. 10.1021/acs.est.7b00508. 28457132 PMC5661887

[ref44] Ahmed A.; Cho Y.; Giles K.; Riches E.; Lee J. W.; Kim H. I.; Choi C. H.; Kim S. Elucidating Molecular Structures of Nonalkylated and Short-Chain Alkyl (*n* < 5, (CH2)n) Aromatic Compounds in Crude Oils by a Combination of Ion Mobility and Ultrahigh-Resolution Mass Spectrometries and Theoretical Collisional Cross-Section Calculations. Anal. Chem. 2014, 86 (7), 3300–3307. 10.1021/ac4032737. 24592806

[ref45] Ibrahim Y. M.; Garimella S. V. B.; Prost S. A.; Wojcik R.; Norheim R. V.; Baker E. S.; Rusyn I.; Smith R. D. Development of an Ion Mobility Spectrometry-Orbitrap Mass Spectrometer Platform. Anal. Chem. 2016, 88 (24), 12152–12160. 10.1021/acs.analchem.6b03027. 28193022 PMC6211177

[ref46] Santos J. M.; Galaverna R. d. S.; Pudenzi M. A.; Schmidt E. M.; Sanders N. L.; Kurulugama R. T.; Mordehai A.; Stafford G. C.; Wisniewski A.; Eberlin M. N. Petroleomics by ion mobility mass spectrometry: resolution and characterization of contaminants and additives in crude oils and petrofuels. Anal. Methods 2015, 7 (11), 4450–4463. 10.1039/C5AY00265F.

[ref47] Schrader W.; Xuan Y.; Gaspar A. Studying ultra-complex crude oil mixtures by using High Field Asymmetric Waveform Ion Mobility Spectrometry (FAIMS) coupled to an ESI-LTQ-Orbitrap Mass Spectrometer. Eur. J. Mass Spectrom. 2014, 20 (1), 43 10.1255/ejms.1263. 24881454

[ref48] Le Maître J.; Hubert-Roux M.; Paupy B.; Marceau S.; Rüger C. P.; Afonso C.; Giusti P. Structural analysis of heavy oil fractions after hydrodenitrogenation by high-resolution tandem mass spectrometry and ion mobility spectrometry. Faraday Discuss. 2019, 218 (0), 417–430. 10.1039/C8FD00239H. 31120046

[ref49] Le Maître J.; Paupy B.; Hubert-Roux M.; Marceau S.; Rüger C.; Afonso C.; Giusti P. Structural Analysis of Neutral Nitrogen Compounds Refractory to the Hydrodenitrogenation Process of Heavy Oil Fractions by High-Resolution Tandem Mass Spectrometry and Ion Mobility-Mass Spectrometry. Energy Fuels 2020, 34 (8), 9328–9338. 10.1021/acs.energyfuels.0c01160.

[ref50] Szykuła K. M.; Wicking C.; Whitmarsh S.; Creaser C. S.; Reynolds J. C. Characterization of Crude Oil and Its Saturate, Aromatic, and Resin Fractions by High-Field Asymmetric Waveform Ion Mobility Spectrometry-High-Resolution Mass Spectrometry. Energy Fuels 2018, 32 (11), 11310–11316. 10.1021/acs.energyfuels.8b02718.

[ref51] Vetere A.; Schrader W. 1-and 2-Photon Ionization for Online FAIMS-FTMS Coupling Allows New Insights into the Constitution of Crude Oils. Anal. Chem. 2015, 87 (17), 8874–8879. 10.1021/acs.analchem.5b01969. 26221748

[ref52] Riedeman J. S.; Kadasala N. R.; Wei A.; Kenttämaa H. I. Characterization of Asphaltene Deposits by Using Mass Spectrometry and Raman Spectroscopy. Energy Fuels 2016, 30 (2), 805–809. 10.1021/acs.energyfuels.5b02002.

[ref53] Tang W.; Hurt M. R.; Sheng H.; Riedeman J. S.; Borton D. J.; Slater P.; Kenttämaa H. I. Structural Comparison of Asphaltenes of Different Origins Using Multi-stage Tandem Mass Spectrometry. Energy Fuels 2015, 29 (3), 1309–1314. 10.1021/ef501242k.

[ref54] Silva L. C. d.; Dávila J. V.; Fleming F. P.; Combariza M. Y.; Vaz B. G. Laser desorption ionization and collision induced dissociastion as powerful tools for FT-ICR mass spectrometric characterization of asphaltene fractions enriched in island and archipelago motifs. Fuel 2022, 323, 124418 10.1016/j.fuel.2022.124418.

[ref55] Barnett D. A.; Ouellette R. J. Elimination of the helium requirement in high-field asymmetric waveform ion mobility spectrometry (FAIMS): beneficial effects of decreasing the analyzer gap width on peptide analysis. Rapid Commun. Mass Spectrom. 2011, 25 (14), 1959–1971. 10.1002/rcm.5078. 21698679

[ref56] Porter D. J.; Mayer P. M.; Fingas M. Analysis of Petroleum Resins Using Electrospray Ionization Tandem Mass Spectrometry. Energy Fuels 2004, 18 (4), 987–994. 10.1021/ef0340099.

[ref57] Vetere A.; Alachraf W.; Panda S. K.; Andersson J. T.; Schrader W. Studying the fragmentation mechanism of selected components present in crude oil by CID mass spectrometry. Rapid Commun. Mass Spectrom. 2018, 32 (24), 2141–2151. 10.1002/rcm.8280. 30198194

[ref58] Bosma N. L.; Harrison A. G. An energy-resolved study of the fragmentation reactions of alkyl ammonium ions. Can. J. Chem. 1994, 72 (11), 2205–2211. 10.1139/v94-281.

[ref59] Peters J.; Clemen M.; Grotemeyer J. Fragmentation of deuterated rhodamine B derivates by laser and collisional activation in an FT-ICR mass spectrometer. Anal. Bioanal. Chem. 2013, 405 (22), 7061–7069. 10.1007/s00216-013-7078-8. 23771505

[ref60] Flego C.; Zannoni C. N-containing species in crude oil fractions: An identification and quantification method by comprehensive two-dimensional gas chromatography coupled with quadrupole mass spectrometry. Fuel 2011, 90 (9), 2863–2869. 10.1016/j.fuel.2011.04.040.

[ref61] Lissitsyna K.; Huertas S.; Quintero L. C.; Polo L. M. Novel simple method for quantitation of nitrogen compounds in middle distillates using solid phase extraction and comprehensive two-dimensional gas chromatography. Fuel 2013, 104, 752–757. 10.1016/j.fuel.2012.08.054.

[ref62] Singh D.; Chopra A.; Patel M. B.; Sarpal A. S. A Comparative Evaluation of Nitrogen Compounds in Petroleum Distillates. Chromatographia 2011, 74 (1), 121–126. 10.1007/s10337-011-2027-1.

