# Supplementary material for: Studying Structural Details in Complex Samples: II. High Field Asymmetric Waveform Ion Mobility Spectrometry (FAIMS) Coupled to High Resolution Tandem Mass Spectrometry (MS/MS)
Source: J Am Soc Mass Spectrom. 2024 Nov 25;36(1):34–43. doi: 10.1021/jasms.4c00227 (PMC11697342; doi:10.1021/jasms.4c00227)
Supplement: Supplementary file 1 — js4c00227_si_001.pdf [file js4c00227_si_001.pdf]

Studying Structural Details in Complex Samples: II. High Field Asymmetric Waveform Ion Mobility Spectrometry (FAIMS) coupled to high resolution tandem mass spectrometry (MS/MS)

Alessandro Vetere, Wolfgang Schrader*

Max-Planck-Institut für Kohlenforschung, Kaiser-Wilhelm-Platz 1, D-45470 Mülheim (Ruhr), Germany

SUPPORTING INFORMATION

Corresponding Author

Wolfgang Schrader - wschrader@kofo.mpg.de

Optimization of ion transmission

The initial FAIMS-FTMS system suffered from one major problem. The commercial Thermo FAIMS unit uses an entrance voltage of 1 kV. This is not an issue when using electrospray ionization where the spray is kept at even higher voltages but it is a problem with ion sources that do not need a high voltage potential for ionization like APPI. Here, the ions have to climb up a (high voltage) hill to reach the entrance of the FAIMS unit which results in a loss of sensitivity. To help them reach their target and improve the sensitivity an additional pusher electrode was installed behind the spray region in a manner that it allows to give the ions a push to better enter the separation unit. The effect of this newly introduced pusher electrode was first evaluated using a set of PAH (polycyclic aromatic hydrocarbons) as model compounds. Figure S1 shows the development of analyte signal (absolute intensities) with increasing pusher electrode potential.

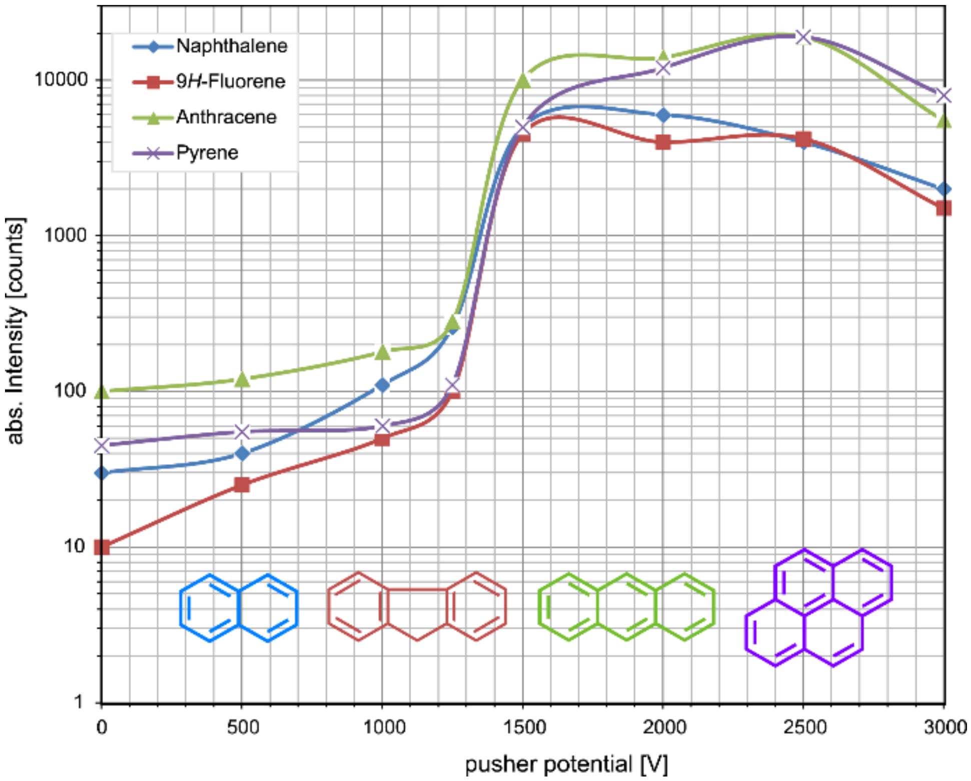

Figure S1. Signal intensities obtained from a PAH standard mixture after APPI-FAIMS-FTMS in relation to the potential applied to the pusher electrode.

The initially low signal intensity is increased by a factor of 2 to 5 when the pusher electrode is operated at the entrance plate potential (1 kV). Further raising the potential to 1.5 kV results in a steep increase in signal intensity by a factor of 100 to 500. Incrementing the potential beyond this point does not enhance the signal, but partly leads to a loss of signal intensity. This behavior is presumably caused by the ions being accelerated too strongly, leading to a collision with the central electrode.

For the remainder of this study a pusher electrode poten-tial of 1.5 kV was thus chosen as the optimum operating condition.

Ion transmission of signals monitored during this study

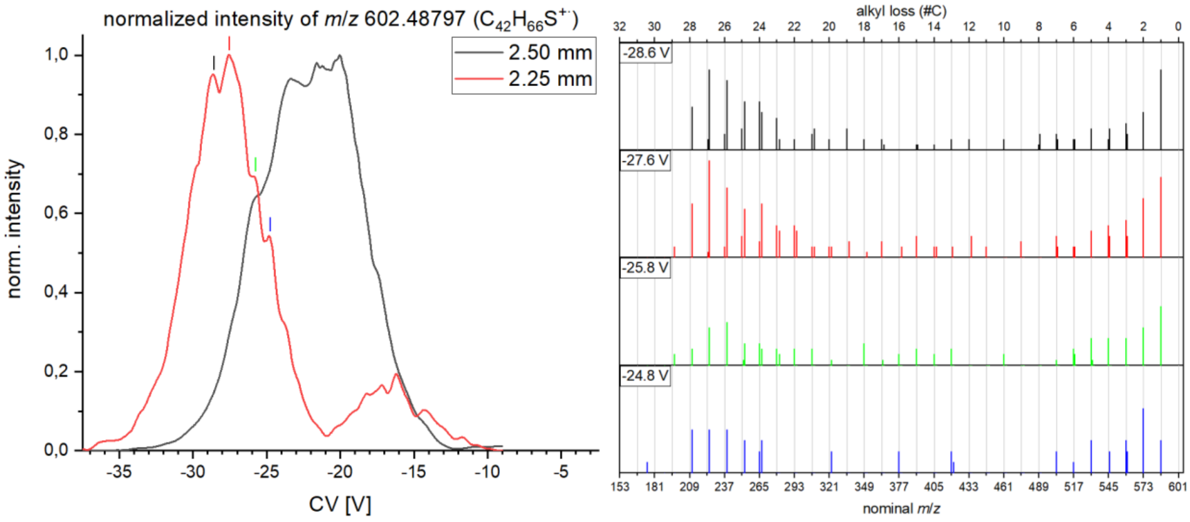

Figure S2. Left: Normalized signal intensity of *m/z* 602.48797 (corresponding to a radical ion of composition $C_{42}H_{66}S^{+\cdot}$) throughout a FAIMS separation using the standard electrodes (black line) and the modified electrodes with smaller gap (red line). Right: Resulting fragment spectra relating to local maxima of the curve.

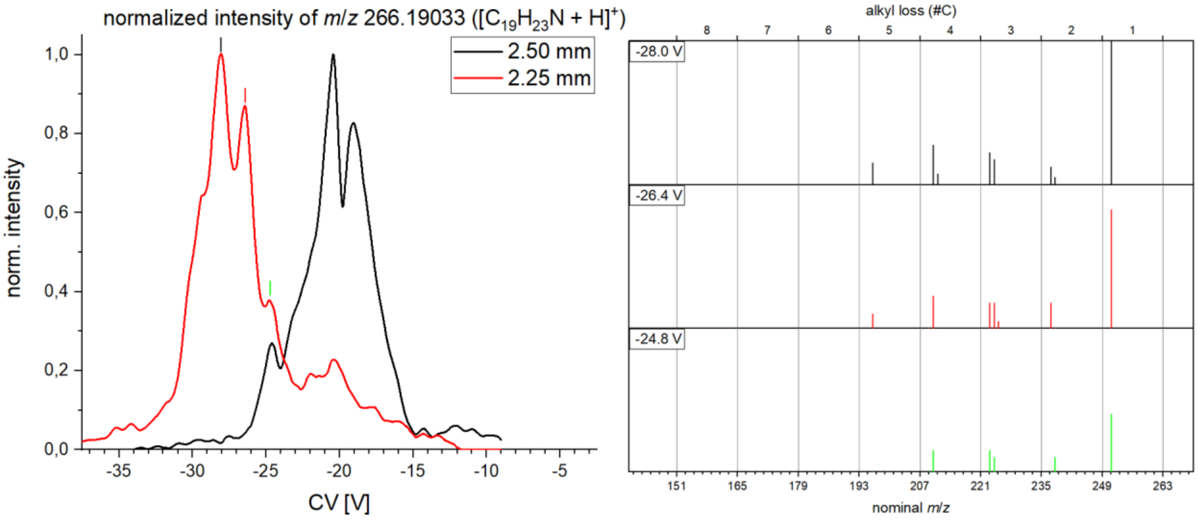

Figure S3. Left: Normalized signal intensity of *m/z* 206.19033 (corresponding to a protonated molecule of composition $[C_{19}H_{23}N + H]^+$) throughout a FAIMS separation using the standard electrodes (black line) and the modified electrodes with smaller gap (red line). Right: Resulting fragment spectra relating to local maxima of the curve.

Isolation window with and without use of FAIMS – Peak annotation

Table S1. Signals identified within the isolation window around *m/z* 602 without using FAIMS (left columns) and with use of FAIMS at a compensation voltage of -29 V (right columns). Center columns indicate peak annotations with elemental compositions.

w/o FAIMS					w. FAIMS (-29V)	
ppm error	*m/z*	Formula	DBE	theor. *m/z*	*m/z*	ppm error
	602.09597					
-0.59	602.09944	$[C_{45}H_{15}NS + H]^+$	38.5	602.099797		
0.22	602.10330	$[C_{42}H_{19}NS_2 + H]^+$	33.5	602.103167		
	602.10700					
-0.29	602.11220	$C_{46}H_{18}S^{+\cdot}$	38.0	602.112373		
-0.52	602.11543	$C_{43}H_{22}S_2^{+\cdot}$	33.0	602.115744		
0.08	602.11916	$C_{40}H_{26}S_3^{+\cdot}$	28.0	602.119114		
-0.14	602.12240	$C_{37}H_{30}S_4^{+\cdot}$	23.0	602.122485		
	602.13685					
	602.14891					
	602.15295					
-0.50	602.15701	$[C_{43}H_{23}NOS + H]^+$	32.5	602.157312		
0.48	602.16097	$[C_{40}H_{27}NOS_2 + H]^+$	27.5	602.160682		
	602.16949					
-0.33	602.17306	$C_{41}H_{30}OS_2^{+\cdot}$	27.0	602.173258		
0.55	602.17696	$C_{38}H_{34}OS_3^{+\cdot}$	22.0	602.176629		
-0.85	602.18061	$C_{43}H_{26}N_2S^{+\cdot}$	32.0	602.181121		
	602.18452					
-0.62	602.18548	$C_{46}{}^{13}C_1H_{23}N^{+\cdot}$	37.0	602.185856		
-0.23	602.19019	$[C_{47}H_{23}N + H]^+$	36.5	602.190326		
-0.24	602.19355	$[C_{44}H_{27}NS + H]^+$	31.5	602.193697		
0.07	602.19711	$[C_{41}H_{31}NS_2 + H]^+$	26.5	602.197068	602.19684	-0.38
	602.20294					
-1.13	602.20559	$C_{45}H_{30}S^{+\cdot}$	31.0	602.206273		
-0.09	602.20959	$C_{42}H_{34}S_2^{+\cdot}$	26.0	602.209644		
0.06	602.21305	$C_{39}H_{38}S_3^{+\cdot}$	21.0	602.213015	602.21319	0.29
0.06	602.21642	$C_{36}H_{42}S_4^{+\cdot}$	16.0	602.216386	602.21655	0.27
-0.15	602.22731	$C_{42}H_{34}O_2S^{+\cdot}$	26.0	602.227402		
0.68	602.23118	$C_{39}H_{38}O_2S_2^{+\cdot}$	21.0	602.230773		
-0.42	602.24759	$[C_{45}H_{31}NO + H]^+$	30.5	602.247841		
0.06	602.25125	$[C_{42}H_{35}NOS + H]^+$	25.5	602.251212		

w/o FAIMS					w. FAIMS (-29V)	
ppm error	*m/z*	Formula	DBE	theor. *m/z*	*m/z*	ppm error
0.28	602.25475	$[C_{39}H_{39}NOS_2 + H]^+$	20.5	602.254583		
-0.53	602.25900	$[C_{42}{}^{13}C_1H_{36}OS + H]^+$	25.5	602.259318		
-0.63	602.26341	$C_{43}H_{38}OS^{+\cdot}$	25.0	602.263788		
-0.16	602.26706	$C_{40}H_{42}OS_2^{+\cdot}$	20.0	602.267159	602.26733	0.28
-0.90	602.27111	$C_{45}H_{34}N_2^{+\cdot}$	30.0	602.271651	602.27118	-0.78
-0.41	602.27504	$[C_{44}{}^{13}C_2H_{33}N + H]^+$	30.5	602.275286		
-1.47	602.27887	$C_{45}{}^{13}C_1H_{35}N^{+\cdot}$	30.0	602.279757		
-0.49	602.28393	$[C_{46}H_{35}N + H]^+$	29.5	602.284227		
-0.51	602.28729	$[C_{43}H_{39}NS + H]^+$	24.5	602.287597	602.28699	-1.01
0.00	602.29097	$[C_{40}H_{43}NS_2 + H]^+$	19.5	602.290968		
-0.48	602.29541	$[C_{43}{}^{13}C_1H_{40}S + H]^+$	24.5	602.2957		
		$[C_{40}{}^{13}C_1H_{44}S_2 + H]^+$	19.5	602.299074	602.29877	-0.51
-0.10	602.30011	$C_{44}H_{42}S^{+\cdot}$	24.0	602.300173		
-0.04	602.30352	$C_{41}H_{46}S_2^{+\cdot}$	19.0	602.303544	602.30365	0.18
0.02	602.30693	$C_{38}H_{50}S_3^{+\cdot}$	14.0	602.306915	602.30713	0.36
		$C_{35}H_{54}S_4^{+\cdot}$	9.0	602.310286	602.31036	0.12
-0.32	602.31774	$C_{44}H_{42}O_2^{+\cdot}$	24.0	602.317932		
-0.04	602.32128	$C_{41}H_{46}O_2S^{+\cdot}$	19.0	602.321303	602.32147	0.28
0.09	602.32473	$C_{38}H_{50}O_2S_2^{+\cdot}$	14.0	602.324674	602.32440	-0.45
0.27	602.32879	$[C_{38}H_{49}NOS_1{}^{34}S_1 + H]^+$	14.5	602.328629		
-0.37	602.33258	$[C_{42}{}^{13}C_2H_{41}NO + H]^+$	24.5	602.332801		
	602.33756					
		$[C_{39}H_{52}OS_1{}^{34}S_1{}^{+\cdot}$	14.0	602.34121	602.34082	-0.65
-0.67	602.34134	$[C_{44}H_{43}NO + H]^+$	23.5	602.341741		
-0.02	602.34510	$[C_{41}H_{47}NOS + H]^+$	18.5	602.345112		
0.29	602.34866	$[C_{38}H_{51}NOS_2 + H]^+$	13.5	602.348483		
-0.26	602.35416	$C_{45}H_{46}O^{+\cdot}$	23.0	602.354318		
-0.11	602.35762	$C_{42}H_{50}OS^{+\cdot}$	18.0	602.357688	602.35773	0.07
0.07	602.36110	$C_{39}H_{54}OS_2^{+\cdot}$	13.0	602.361059	602.36114	0.13
-0.57	602.36521	$C_{44}H_{46}N_2^{+\cdot}$	23.0	602.365551		
0.11	602.36925	$[C_{43}{}^{13}C_2H_{45}N + H]^+$	23.5	602.369187		
0.01	602.37366	$C_{44}{}^{13}C_1H_{47}N^{+\cdot}$	23.0	602.373657		
-0.83	602.37763	$[C_{45}H_{47}N + H]^+$	22.5	602.378127	602.37781	-0.53
0.04	602.38152	$[C_{42}H_{51}NS + H]^+$	17.5	602.381498	602.38135	-0.25

w/o FAIMS					w. FAIMS (-29V)	
ppm error	*m/z*	Formula	DBE	theor. *m/z*	*m/z*	ppm error
0.38	602.38536	$C_{41}{}^{13}C_2H_{52}S^{+\cdot}$	18.0	602.385134	602.38470	-0.72
		$C_{38}{}^{13}C_2H_{56}S_2^{+\cdot}$	13.0	602.3885	602.38867	0.28
-0.35	602.39049	$C_{46}H_{50}^{+\cdot}$	22.0	602.390703		
-0.01	602.39407	$C_{43}H_{54}S^{+\cdot}$	17.0	602.394074	602.39422	0.24
0.02	602.39746	$C_{40}H_{58}S_2^{+\cdot}$	12.0	602.397445	602.39746	0.02
0.35	602.40310	$C_{41}{}^{13}C_2H_{52}O_2^{+\cdot}$	18.0	602.402892		
	602.40677					
-0.37	602.41161	$C_{43}H_{54}O_2^{+\cdot}$	17.0	602.411832	602.41180	-0.05
-0.12	602.41513	$C_{40}H_{58}O_2S^{+\cdot}$	12.0	602.415203	602.41510	-0.17
0.06	602.41861	$C_{37}H_{62}O_2S_2^{+\cdot}$	7.0	602.418574		
0.09	602.42312	$C_{42}H_{54}N_2O^{+\cdot}$	17.0	602.423066		
-0.27	602.42654	$[C_{41}{}^{13}C_2H_{53}NO + H]^+$	17.5	602.426701		
0.08	602.43122	$C_{42}{}^{13}C_1H_{55}NO^{+\cdot}$	17.0	602.431172	602.43115	-0.04
-0.02	602.43563	$[C_{43}H_{55}NO + H]^+$	16.5	602.435642		
0.36	602.43923	$[C_{40}H_{59}NOS + H]^+$	11.5	602.439013		
		$C_{39}{}^{13}C_2H_{60}OS^{+\cdot}$	12.0	602.44265	602.44293	0.46
-0.35	602.44801	$C_{44}H_{58}O^{+\cdot}$	16.0	602.448218	602.44824	0.04
-0.05	602.45156	$C_{41}H_{62}OS^{+\cdot}$	11.0	602.451589	602.45172	0.22
0.06	602.45502	$[C_{42}{}^{13}C_1H_{56}N_2 + H]^+$	16.5	602.454981		
0.20	602.45957	$C_{43}H_{58}N_2^{+\cdot}$	16.0	602.459451	602.45923	-0.37
0.49	602.46338	$[C_{42}{}^{13}C_2H_{57}N + H]^+$	16.5	602.463087		
0.17	602.46766	$C_{43}{}^{13}C_1H_{59}N^{+\cdot}$	16.0	602.467557	602.46777	0.35
-0.04	602.47200	$[C_{44}H_{59}N + H]^+$	15.5	602.472027	602.47229	0.44
-0.20	602.47554	$C_{43}{}^{13}C_2H_{60}+\cdot$	16.0	602.475663	602.47558	-0.14
0.59	602.47939	$C_{40}{}^{13}C_2H_{64}S^{+\cdot}$	11.0	602.479034	602.47912	0.14
0.00	602.48460	$C_{45}H_{62}^{+\cdot}$	15.0	602.484603	602.48474	0.23
-0.01	602.48797	$C_{42}H_{66}S^{+\cdot}$	10.0	602.487974	602.48797	-0.01
0.09	602.49140	$C_{39}H_{70}S_2^{+\cdot}$	5.0	602.491345	602.49121	-0.22
0.06	602.50130	$[C_{41}{}^{13}C_1H_{64}O_2 + H]^+$	10.5	602.501263		
0.34	602.50594	$C_{42}H_{66}O_2^{+\cdot}$	10.0	602.505733	602.50543	-0.50
0.14	602.50919	$C_{39}H_{70}O_2S^{+\cdot}$	5.0	602.509104		
	602.52255					
0.26	602.52523	$C_{41}{}^{13}C_1H_{67}NO^{+\cdot}$	10.0	602.525072		
0.16	602.52964	$[C_{42}H_{67}NO + H]^+$	9.5	602.529542		

w/o FAIMS					w. FAIMS (-29V)	
ppm error	*m/z*	Formula	DBE	theor. *m/z*	*m/z*	ppm error
		$C_{41}{}^{13}C_2H_{68}O^{+\cdot}$	10.0	602.53318	602.53338	0.33
0.22	602.53778	$[C_{42}{}^{13}C_1H_{68}O + H]^+$	9.5	602.537648	602.53784	0.32
-0.18	602.54201	$C_{43}H_{70}O^{+\cdot}$	9.0	602.542118	602.54211	-0.01
0.60	602.54585	$C_{40}H_{74}OS^{+\cdot}$	4.0	602.545489		
	602.55093					
0.15	602.55708	$[C_{41}{}^{13}C_2H_{69}N + H]^+$	9.5	602.556987		
0.07	602.56150	$C_{42}{}^{13}C_1H_{71}N^{+\cdot}$	9.0	602.561458	602.56158	0.20
0.04	602.56595	$[C_{43}H_{71}N + H]^+$	8.5	602.565928	602.56586	-0.11
0.08	602.56961	$C_{42}{}^{13}C_2H_{72}^{+\cdot}$	9.0	602.569563	602.56940	-0.27
-0.01	602.57403	$[C_{43}{}^{13}C_1H_{72} + H]^+$	8.5	602.574034	602.57391	-0.21
0.03	602.57852	$C_{44}H_{74}^{+\cdot}$	8.0	602.578504	602.57855	0.08
0.22	602.58201	$C_{41}H_{78}S^{+\cdot}$	3.0	602.581875	602.58185	-0.04

Table S2. Signals identified from the MS² scan of *m/z* 602 with use of FAIMS at a compensation voltage of -29 V. Comments indicate the reason for identifying the fragment as belonging to a co-isolated compound and excluding it from interpretation (to high amount of heteroatoms or unreasonable DBE). Here, 46 of the 149 fragment ions were used to for the interpretation of the CV voltage of -29 V.

m/z	Composition	DBE	theor. *m/z*	ppm error	comment
175.05776	$[C_{11}H_{10}S + H]^+$	6.5	175.0576	0.95	
211.05789	$[C_{14}H_{10}S + H]^+$	9.5	211.0576	1.38	
212.06563	$C_{14}H_{12}S^{+\cdot}$	9.0	212.06542	0.97	
225.07342	$[C_{15}H_{12}S + H]^+$	9.5	225.07325	0.76	
226.0812	$C_{15}H_{14}S^{+\cdot}$	9.0	226.08107	0.58	
237.07344	$[C_{16}H_{12}S + H]^+$	10.5	237.07325	0.81	
239.08907	$[C_{16}H_{14}S + H]^+$	9.5	239.0889	0.72	
240.09684	$C_{16}H_{16}S^{+\cdot}$	9.0	240.09672	0.49	
251.08894	$[C_{17}H_{14}S + H]^+$	10.5	251.0889	0.16	
253.1046	$[C_{17}H_{16}S + H]^+$	9.5	253.10455	0.21	
263.76047					
265.10456	$[C_{18}H_{16}S + H]^+$	10.5	265.10455	0.05	
267.02986	$[C_{16}H_{10}S_2 + H]^+$	11.5	267.02967	0.72	# of heteroatoms
267.12031	$[C_{18}H_{18}S + H]^+$	9.5	267.1202	0.43	
269.04549	$[C_{16}H_{12}S_2 + H]^+$	10.5	269.04532	0.64	# of heteroatoms
279.12026	$[C_{19}H_{18}S + H]^+$	10.5	279.1202	0.22	
281.04537	$[C_{17}H_{12}S_2 + H]^+$	11.5	281.04532	0.2	# of heteroatoms

m/z	Composition	DBE	theor. m/z	ppm error	comment
281.13269	$[C_{22}H_{16} + H]^+$	14.5	281.13248	0.74	too high DBE
281.13595	$[C_{19}H_{20}S + H]^+$	9.5	281.13585	0.37	
282.05328	$C_{17}H_{14}S_2^{+\cdot}$	11.0	282.05314	0.49	# of heteroatoms
293.09955	$[C_{19}H_{16}OS + H]^+$	11.5	293.09946	0.29	# of heteroatoms
293.13263	$[C_{23}H_{16} + H]^+$	15.5	293.13248	0.52	too high DBE
293.13596	$[C_{20}H_{20}S + H]^+$	10.5	293.13585	0.37	
295.06092	$[C_{18}H_{14}S_2 + H]^+$	11.5	295.06097	-0.17	# of heteroatoms
295.15153	$[C_{20}H_{22}S + H]^+$	9.5	295.1515	0.1	
296.06886	$C_{18}H_{16}S_2^{+\cdot}$	11.0	296.06879	0.21	# of heteroatoms
307.06108	$[C_{19}H_{14}S_2 + H]^+$	12.5	307.06097	0.36	# of heteroatoms
307.14833	$[C_{24}H_{18} + H]^+$	15.5	307.14813	0.65	too high DBE
307.15156	$[C_{21}H_{22}S + H]^+$	10.5	307.1515	0.21	
309.07668	$[C_{19}H_{16}S_2 + H]^+$	11.5	309.07662	0.19	# of heteroatoms
309.16384	$[C_{24}H_{20} + H]^+$	14.5	309.16378	0.19	too high DBE
309.16719	$[C_{21}H_{24}S + H]^+$	9.5	309.16715	0.15	
310.08446	$C_{19}H_{18}S_2^{+\cdot}$	11.0	310.08444	0.06	# of heteroatoms
311.08889	$[C_{22}H_{14}S + H]^+$	15.5	311.0889	-0.02	too high DBE
315.12025	$[C_{22}H_{18}S + H]^+$	13.5	315.1202	0.15	too high DBE
321.07668	$[C_{20}H_{16}S_2 + H]^+$	12.5	321.07662	0.2	# of heteroatoms
321.16726	$[C_{22}H_{24}S + H]^+$	10.5	321.16715	0.35	
323.09235	$[C_{20}H_{18}S_2 + H]^+$	11.5	323.09227	0.24	# of heteroatoms
323.17963	$[C_{25}H_{22} + H]^+$	14.5	323.17943	0.62	too high DBE
323.18292	$[C_{22}H_{26}S + H]^+$	9.5	323.1828	0.38	
335.09223	$[C_{21}H_{18}S_2 + H]^+$	12.5	335.09227	-0.12	# of heteroatoms
335.1829	$[C_{23}H_{26}S + H]^+$	10.5	335.1828	0.3	
337.10795	$[C_{21}H_{20}S_2 + H]^+$	11.5	337.10792	0.1	# of heteroatoms
337.19514	$[C_{26}H_{24} + H]^+$	14.5	337.19508	0.19	too high DBE
337.19853	$[C_{23}H_{28}S + H]^+$	9.5	337.19845	0.24	
338.11269	$C_{24}H_{18}S^{+\cdot}$	16.0	338.11237	0.92	too high DBE
338.2029	$C_{26}H_{26}^{+\cdot}$	14.0	338.2029	-0.02	too high DBE
349.10787	$[C_{22}H_{20}S_2 + H]^+$	12.5	349.10792	-0.15	# of heteroatoms
349.1588	$[C_{26}H_{20}O + H]^+$	16.5	349.15869	0.31	# of heteroatoms
349.19866	$[C_{24}H_{28}S + H]^+$	10.5	349.19845	0.6	
351.12032	$[C_{25}H_{18}S + H]^+$	16.5	351.1202	0.36	too high DBE
351.12365	$[C_{22}H_{22}S_2 + H]^+$	11.5	351.12357	0.23	# of heteroatoms
351.21429	$[C_{24}H_{30}S + H]^+$	9.5	351.2141	0.54	
363.12027	$[C_{26}H_{18}S + H]^+$	17.5	363.1202	0.19	too high DBE
363.12356	$[C_{23}H_{22}S_2 + H]^+$	12.5	363.12357	-0.01	# of heteroatoms
363.21414	$[C_{25}H_{30}S + H]^+$	10.5	363.2141	0.12	
365.13587	$[C_{26}H_{20}S + H]^+$	16.5	365.13585	0.06	too high DBE
365.13923	$[C_{23}H_{24}S_2 + H]^+$	11.5	365.13922	0.02	# of heteroatoms

m/z	Composition	DBE	theor. *m/z*	ppm error	comment
365.22986	$[C_{25}H_{32}S + H]^+$	9.5	365.22975	0.31	
377.04878	$[C_{22}H_{16}S_3 + H]^+$	14.5	377.04869	0.23	# of heteroatoms
377.13587	$[C_{27}H_{20}S + H]^+$	17.5	377.13585	0.07	too high DBE
377.13923	$[C_{24}H_{24}S_2 + H]^+$	12.5	377.13922	0.03	# of heteroatoms
377.22981	$[C_{26}H_{32}S + H]^+$	10.5	377.22975	0.16	
378.23762	$C_{26}H_{34}S^{+\cdot}$	10.0	378.23757	0.13	
391.06451	$[C_{23}H_{18}S_3 + H]^+$	14.5	391.06434	0.42	# of heteroatoms
391.1184	$[C_{24}H_{22}OS_2 + H]^+$	13.5	391.11848	-0.21	# of heteroatoms
391.15179	$[C_{28}H_{22}S + H]^+$	17.5	391.1515	0.74	too high DBE
391.15481	$[C_{25}H_{26}S_2 + H]^+$	12.5	391.15487	-0.16	# of heteroatoms
391.2419	$[C_{30}H_{30} + H]^+$	15.5	391.24203	-0.31	too high DBE
391.24541	$[C_{27}H_{34}S + H]^+$	10.5	391.2454	0.04	
393.17056	$[C_{25}H_{28}S_2 + H]^+$	11.5	393.17052	0.11	# of heteroatoms
405.16727	$[C_{29}H_{24}S + H]^+$	17.5	405.16715	0.3	too high DBE
405.17057	$[C_{26}H_{28}S_2 + H]^+$	12.5	405.17052	0.12	# of heteroatoms
405.25772	$[C_{31}H_{32} + H]^+$	15.5	405.25768	0.12	too high DBE
405.26102	$[C_{28}H_{36}S + H]^+$	10.5	405.26105	-0.07	
419.183	$[C_{30}H_{26}S + H]^+$	17.5	419.1828	0.48	too high DBE
419.18629	$[C_{27}H_{30}S_2 + H]^+$	12.5	419.18617	0.3	# of heteroatoms
419.27678	$[C_{29}H_{38}S + H]^+$	10.5	419.2767	0.19	
433.10809	$[C_{29}H_{20}S_2 + H]^+$	19.5	433.10792	0.39	# of heteroatoms
433.20175	$[C_{28}H_{32}S_2 + H]^+$	12.5	433.20182	-0.16	# of heteroatoms
433.29257	$[C_{30}H_{40}S + H]^+$	10.5	433.29235	0.51	
447.21424	$[C_{32}H_{30}S + H]^+$	17.5	447.2141	0.32	too high DBE
461.32369	$[C_{32}H_{44}S + H]^+$	10.5	461.32365	0.09	
475.2486	$[C_{31}H_{38}S_2 + H]^+$	12.5	475.24877	-0.36	# of heteroatoms
478.3628	$C_{33}H_{50}S^{+\cdot}$	9.0	478.36277	0.06	
489.26445	$[C_{32}H_{40}S_2 + H]^+$	12.5	489.26442	0.06	# of heteroatoms
490.36308	$C_{34}H_{50}S^{+\cdot}$	10.0	490.36277	0.63	
503.27996	$[C_{33}H_{42}S_2 + H]^+$	12.5	503.28007	-0.21	# of heteroatoms
503.37072	$[C_{35}H_{50}S + H]^+$	10.5	503.3706	0.25	
504.3786	$C_{35}H_{52}S^{+\cdot}$	10.0	504.37842	0.35	
517.29594	$[C_{34}H_{44}S_2 + H]^+$	12.5	517.29572	0.42	# of heteroatoms
517.38608	$[C_{36}H_{52}S + H]^+$	10.5	517.38625	-0.32	
518.39408	$C_{36}H_{54}S^{+\cdot}$	10.0	518.39407	0.02	
531.31105	$[C_{35}H_{46}S_2 + H]^+$	12.5	531.31137	-0.6	# of heteroatoms
531.40187	$[C_{37}H_{54}S + H]^+$	10.5	531.4019	-0.05	
532.31917	$C_{35}H_{48}S_2^{+\cdot}$	12.0	532.31919	-0.04	# of heteroatoms
545.32385	$[C_{39}H_{44}S + H]^+$	17.5	545.32365	0.37	too high DBE
545.32703	$[C_{36}H_{48}S_2 + H]^+$	12.5	545.32702	0.01	# of heteroatoms

m/z	Composition	DBE	theor. *m/z*	ppm error	comment
545.39709	$[C_{39}{}^{13}C_1H_{49}N + H]^+$	16.5	545.39713	-0.07	# of heteroatoms
545.41785	$[C_{38}H_{56}S + H]^+$	10.5	545.41755	0.55	
546.3349	$C_{36}H_{50}S_2^{+\cdot}$	12.0	546.33484	0.11	# of heteroatoms
546.40485	$C_{39}{}^{13}C_1H_{51}N^{+\cdot}$	16.0	546.60696	-0.2	# of heteroatoms
546.42536	$C_{38}H_{58}S^{+\cdot}$	10.0	546.42537	-0.03	
559.33952	$[C_{40}H_{46}S + H]^+$	17.5	559.3393	0.39	too high DBE
559.34251	$[C_{37}H_{50}S_2 + H]^+$	12.5	559.34267	-0.29	# of heteroatoms
559.41288	$[C_{40}{}^{13}C_1H_{51}N + H]^+$	16.5	559.41278	0.18	# of heteroatoms
559.42955	$[C_{42}H_{54} + H]^+$	15.5	559.42983	-0.5	too high DBE
559.43309	$[C_{39}H_{58}S + H]^+$	10.5	559.4332	-0.2	
573.3547	$[C_{41}H_{48}S + H]^+$	17.5	573.35495	-0.44	too high DBE
573.35799	$[C_{38}H_{52}S_2 + H]^+$	12.5	573.35832	-0.57	# of heteroatoms
573.42806	$[C_{41}{}^{13}C_1H_{53}N + H]^+$	16.5	573.42843	-0.64	# of heteroatoms
573.44479	$[C_{43}H_{56} + H]^+$	15.5	573.44548	-1.21	too high DBE
573.4482	$[C_{40}H_{60}S + H]^+$	10.5	573.44885	-1.12	
587.28315	$[C_{37}H_{46}S_3 + H]^+$	14.5	587.28344	-0.5	# of heteroatoms
587.33429	$[C_{41}H_{46}OS + H]^+$	18.5	587.33421	0.14	# of heteroatoms
587.35364	$C_{44}H_{45}N^{+\cdot}$	23.0	587.35465	-1.72	# of heteroatoms
587.37049	$[C_{42}H_{50}S + H]^+$	17.5	587.3706	-0.19	too high DBE
587.37366	$[C_{39}H_{54}S_2 + H]^+$	12.5	587.37397	-0.52	# of heteroatoms
587.42475	$[C_{43}H_{54}O + H]^+$	16.5	587.42474	0.01	# of heteroatoms
587.44385	$[C_{42}{}^{13}C_1H_{55}N + H]^+$	16.5	587.44408	-0.39	# of heteroatoms
587.45197	$C_{40}H_{61}NS^{+\cdot}$	11.0	587.45192	0.08	# of heteroatoms
587.45533	$C_{37}H_{65}NS_2^{+\cdot}$	6.0	587.45529	0.06	# of heteroatoms
587.46076	$[C_{44}H_{58} + H]^+$	15.5	587.46113	-0.63	too high DBE
587.46412	$[C_{41}H_{62}S + H]^+$	10.5	587.4645	-0.65	
602.29088	$[C_{40}H_{43}NS_2 + H]^+$	19.5	602.29097	-0.14	# of heteroatoms
602.29967	$C_{44}H_{42}S^{+\cdot}$	24.0	602.30017	-0.83	too high DBE
602.30339	$C_{41}H_{46}S_2^{+\cdot}$	19.0	602.30354	-0.25	# of heteroatoms
602.30681	$C_{38}H_{50}S_3^{+\cdot}$	14.0	602.30692	-0.17	# of heteroatoms
602.35759	$C_{42}H_{50}OS^{+\cdot}$	18.0	602.35769	-0.16	# of heteroatoms
602.37755	$[C_{45}H_{47}N + H]^+$	22.5	602.37813	-0.95	# of heteroatoms
602.3814	$[C_{42}H_{51}NS + H]^+$	17.5	602.3815	-0.17	# of heteroatoms
602.38488	$[C_{39}H_{55}NS_2 + H]^+$	12.5	602.38487	0.01	# of heteroatoms
602.3908	$C_{46}H_{50}^{+\cdot}$	22.0	602.3907	0.16	# of heteroatoms
602.39403	$C_{43}H_{54}S^{+\cdot}$	17.0	602.39407	-0.07	# of heteroatoms
602.39733	$C_{40}H_{58}S_2^{+\cdot}$	12.0	602.39744	-0.19	# of heteroatoms
602.41155	$C_{43}H_{54}O_2^{+\cdot}$	17.0	602.41183	-0.47	# of heteroatoms
602.41845	$C_{37}H_{62}O_2S_2^{+\cdot}$	7.0	602.41857	-0.21	# of heteroatoms
602.43554	$[C_{43}H_{55}ON + H]^+$	16.5	602.43564	-0.18	# of heteroatoms
602.43883	$[C_{40}H_{59}ONS + H]^+$	11.5	602.43901	-0.3	# of heteroatoms
602.44793	$C_{44}H_{58}O^{+\cdot}$	16.0	602.44822	-0.48	# of heteroatoms

m/z	Composition	DBE	theor. *m/z*	ppm error	comment
602.45147	$C_{41}H_{62}OS^{+\cdot}$	11.0	602.45159	-0.2	# of heteroatoms
602.463	$[C_{42}{}^{13}C_2H_{57}N + H]^+$	16.5	602.46309	-0.14	# of heteroatoms
602.46752	$C_{43}{}^{13}C_1H_{59}N^{+\cdot}$	16.0	602.46756	-0.06	# of heteroatoms
602.47197	$[C_{44}H_{59}N + H]^+$	15.5	602.47203	-0.09	# of heteroatoms
602.47533	$[C_{41}H_{63}NS + H]^+$	10.5	602.4754	-0.11	# of heteroatoms
602.48424	$C_{45}H_{62}^{+\cdot}$	15.0	602.4846	-0.6	# of heteroatoms
602.48772	$C_{42}H_{66}S^{+\cdot}$	10.0	602.48797	-0.42	
602.52916	$[C_{42}H_{67}ON + H]^+$	9.5	602.52954	-0.63	# of heteroatoms
602.56566	$[C_{43}H_{71}N + H]^+$	8.5	602.56593	-0.44	# of heteroatoms

Table S3. Signals identified within the isolation window around *m/z* 266 without using FAIMS (left columns) and with use of FAIMS at a compensation voltage of -26.4 V (right columns). Center columns indicate peak annotations with elemental compositions.

w/o FAIMS					w. FAIMS (-26.4V)	
ppm error	*m/z*	Formula	DBE	theor. *m/z*	*m/z*	ppm error
	266.00192					
1.04	266.01318	$C_{14}{}^{13}C_2H_8S_2^{+\cdot}$	13.0	266.01290		
0.02	266.01401	$[C_{18}{}^{13}C_1H_{\$}S + H]^+$	17.5	266.01400		
-0.55	266.01496	$C_{22}H_2^{+\cdot}$	22.0	266.01510		
-0.17	266.01733	$[C_{15}{}^{13}C_1H_8S_2 + H]^+$	12.5	266.01737		
-0.60	266.01831	$C_{19}H_6S^{+\cdot}$	17.0	266.01847		
					266.01931	
-0.22	266.02179	$C_{16}H_{10}S_2^{+\cdot}$	12.0	266.02184	266.02175	-0.36
					266.02306	
-0.24	266.03954	$C_{16}H_{10}O_2S^{+\cdot}$	12.0	266.03960		
	266.04048					
0.25	266.05608	$[C_{14}H_7O_3N_3 + H]^+$	12.5	266.05602		
		$C_{13}H_{14}O_4S^{+\cdot}$	7.0	266.06073	266.06093	0.76
-0.15	266.06337	$[C_{16}H_{11}ONS + H]^+$	11.5	266.06341		
		$[C_{13}H_{15}ONS_2 + H]^+$	6.5	266.06678	266.06676	-0.08
-0.24	266.07145	$[C_{16}{}^{13}C_1H_{12}OS + H]^+$	11.5	266.07152		
-0.03	266.07261	$C_{20}H_{10}O^{+\cdot}$	16.0	266.07262		
-0.40	266.07588	$C_{17}H_{14}OS^{+\cdot}$	11.0	266.07599	266.07589	-0.38
-0.18	266.08380	$C_{19}H_{10}N_2^{+\cdot}$	16.0	266.08385		
0.23	266.09202	$C_{19}{}^{13}C_1H_{11}N^{+\cdot}$	16.0	266.09196		
-0.86	266.09352	$C_{17}H_{14}O_3^{+\cdot}$	11.0	266.09375		
-0.05	266.09641	$[C_{20}H_{11}N + H]^+$	15.5	266.09643	266.09630	-0.46
-0.24	266.09973	$[C_{17}H_{15}NS + H]^+$	10.5	266.09980	266.09966	-0.51
0.12	266.10456	$[C_{20}{}^{13}C_1H_{12} + H]^+$	15.5	266.10453	266.10442	-0.42
0.19	266.10795	$[C_{17}C_1H_{16}S + H]^+$	10.5	266.10790	266.10784	-0.24
-0.03	266.10899	$C_{21}H_{14}^{+\cdot}$	15.0	266.10900	266.10882	-0.70

w/o FAIMS					w. FAIMS (-26.4V)	
ppm error	*m/z*	Formula	DBE	theor. *m/z*	*m/z*	ppm error
-0.21	266.11232	$C_{18}H_{18}S^{+\cdot}$	10.0	266.11237	266.11232	-0.18
-0.59	266.11559	$C_{15}H_{22}S_2^{+\cdot}$	5.0	266.11574	266.11559	-0.58
-0.47	266.11743	$[C_{17}H_{15}O_2N + H]^+$	10.5	266.11756		
-0.24	266.12560	$[C_{17}{}^{13}C_1H_{16}O_2 + H]^+$	10.5	266.12566		
-0.41	266.13002	$C_{18}H_{18}O_2^{+\cdot}$	10.0	266.13013	266.12996	-0.63
-0.15	266.13346	$C_{15}H_{22}O_2S^{+\cdot}$	5.0	266.13350		
-0.68	266.14929	$C_{17}{}^{13}C_1H_{19}ON^{+\cdot}$	10.0	266.14947		
		$C_{15}H_{22}O_4^{+\cdot}$	5.0	266.15126	266.15117	-0.33
-0.27	266.15387	$[C_{18}H_{19}ON + H]^+$	9.5	266.15394	266.15374	-0.77
0.28	266.15739	$[C_{15}H_{23}ONS + H]^+$	4.5	266.15731		
-0.33	266.16196	$[C_{18}{}^{13}C_1H_{20}O + H]^+$	9.5	266.16205	266.16195	-0.38
-0.13	266.16538	$[C_{15}{}^{13}C_1H_{24}OS + H]^+$	4.5	266.16542		
-0.16	266.16647	$C_{19}H_{22}O^{+\cdot}$	9.0	266.16652	266.16643	-0.32
-0.15	266.16985	$C_{16}H_{26}OS^{+\cdot}$	4.0	266.16989	266.16967	-0.83
	266.17305					
-0.12	266.17504	$[C_{15}H_{23}O_3N + H]^+$	4.5	266.17507	266.17489	-0.69
-0.51	266.17761	$C_{18}H_{22}N_2^{+\cdot}$	9.0	266.17775	266.17772	-0.10
-0.44	266.17832	$[C_{12}H_{27}O_3NS + H]^+$	-0.5	266.17844		
-0.23	266.18311	$[C_{15}{}^{13}C_1H_{24}O_3 + H]^+$	4.5	266.18318	266.18297	-0.76
-0.40	266.18575	$C_{18}{}^{13}C_1H_{23}N^{+\cdot}$	9.0	266.18586	266.18569	-0.63
0.57	266.1878	$C_{16}H_{26}O_3^{+\cdot}$	4.0	266.18765	266.18749	-0.59
-0.25	266.19026	$[C_{19}H_{23}N + H]^+$	8.5	266.19033	266.19024	-0.34
0.70	266.19388	$[C_{16}H_{27}NS + H]^+$	3.5	266.19370	266.19381	0.41
-0.23	266.19837	$[C_{19}{}^{13}C_1H_{24} + H]^+$	8.5	266.19843	266.19841	-0.06
0.25	266.20187	$[C_{16}{}^{13}C_1H_{28}S + H]^+$	3.5	266.20180		
-0.03	266.20290	$C_{20}H_{26}^{+\cdot}$	8.0	266.20290	266.20290	-0.01
-0.50	266.20614	$C_{17}H_{30}S^{+\cdot}$	3.0	266.20627	266.20614	-0.52
		$C_{17}H_{30}O_2^{+\cdot}$	3.0	266.22403	266.22393	-0.39
0.01	266.25595	$[C_{17}{}^{13}C_1H_{32}O + H]^+$	2.5	266.25595	266.25576	-0.71
		$C_{18}H_{34}O^{+\cdot}$	2.0	266.26042	266.26030	-0.42
-0.91	266.29656	$C_{19}H_{38}^{+\cdot}$	1.0	266.29680		

Table S4. Signals identified from the MS² scan of *m/z* 266 with use of FAIMS at a compensation voltage of -26.4 V. Comments indicate the reason for identifying the fragment as belonging to a co-isolated compound and excluding it from interpretation (to high amount of heteroatoms/isotopes or unreasonable DBE). Fragment ions marked in green are considered to be ambiguous as they could belong to the desired analyte precursor or to a co-isolated compound and are therefore not considered for interpretation. Here, 9 of the 71 ions present were used for the interpretation of the CV voltage of -26.4 V.

m/z	Composition	DBE	theor. *m/z*	ppm error	comment
88.07566	$[C_4H_9ON + H]^+$	0.5	88.07569	-0.40	# of heteroatoms
150.96628	$C_3{}^{13}C_1H_6S_3{}^{+\cdot}$	2.0	150.96597	2.09	# of heteroatoms
167.99366	$[C_7H5NS_2 + H]^+$	5.5	167.99362	0.28	# of heteroatoms
175.05764	$[C_{11}H_{10}S + H]^+$	6.5	175.05760	0.22	# of heteroatoms
181.10122	$[C_{14}H_{12} + H]^+$	8.5	181.10118	0.25	ambiguous
195.11691	$[C_{15}H_{15}$	8.5	195.11683	0.42	ambiguous
196.11210	$[C_{14}H_{13}N + H]^+$	8.5	196.11208	0.14	
196.12025	$[C_{14}{}^{13}C_1H_{14} + H]^+$	8.5	196.12018	0.36	isotopologue
196.12475	$C_{15}H_{16}{}^{+\cdot}$	8.0	196.12465	0.51	ambiguous
197.13247	$[C_{15}H_{16} + H]^+$	7.5	197.13248	-0.01	ambiguous
209.09610	$[C_{15}H_{12}O + H]^+$	9.5	209.09609	0.02	# of heteroatoms
209.13243	$[C_{16}H_{16} + H]^+$	8.5	209.13248	-0.24	ambiguous
210.10397	$C_{15}H_{14}O^{+\cdot}$	9.0	210.10392	0.25	# of heteroatoms
210.12770	$[C_{15}H_{15}N + H]^+$	8.5	210.12773	-0.14	
210.13577	$[C_{15}{}^{13}C_1H_{16} + H]^+$	8.5	210.13583	-0.30	isotopologue
210.14027	$C_{16}H_{18}{}^{+\cdot}$	8.0	210.14030	-0.15	ambiguous
211.05761	$[C_{14}H_{10}S + H]^+$	9.5	211.05760	0.08	# of heteroatoms
221.04198	$[C_{15}H_8S + H]^+$	11.5	221.04195	0.17	# of heteroatoms
222.03475	$C_{11}H_{10}O_3S^{+\cdot}$	7.0	222.03452	1.07	# of heteroatoms
222.12774	$[C_{16}H_{15}N + H]^+$	9.5	222.12773	0.06	
223.05766	$[C_{15}H_{10}S + H]^+$	10.5	223.05760	0.27	
223.11164	$[C_{16}H_{14}O + H]^+$	9.5	223.11174	-0.44	# of heteroatoms
223.13110	$[C_{15}{}^{13}C_1H_{15}N + H]^+$	9.5	223.13108	0.08	isotopologue
223.13566	$C_{16}H_{17}N^{+\cdot}$	9.0	223.13555	0.49	
223.14808	$[C_{17}H_{18} + H]^+$	8.5	223.14813	-0.21	ambiguous
224.06102	$[C_{14}{}^{13}C_1H_{10}S + H]^+$	10.5	224.06095	0.28	# of heteroatoms
224.06552	$C_{15}H_{12}S^{+\cdot}$	10.0	224.06542	0.42	# of heteroatoms
224.11531	$[C_{15}{}^{13}C_1H_{14}O + H]^+$	9.5	224.11510	0.94	# of heteroatoms
224.11958	$C_{16}H_{16}O^{+\cdot}$	9.0	224.11957	0.05	# of heteroatoms
224.14332	$[C_{16}H_{17}N + H]^+$	8.5	224.14338	-0.24	
224.15155	$[C_{16}{}^{13}C_1H_{18} + H]^+$	8.5	224.15148	0.29	isotopologue
224.15582	$C_{17}H_{20}{}^{+\cdot}$	8.0	224.15595	-0.60	ambiguous
233.12798	$C_{17}{}^{13}C_1H_{16}{}^{+\cdot}$	11.0	233.12801	-0.11	too high DBE
233.13247	$[C_{18}H_{16} + H]^+$	10.5	233.13248	-0.05	too high DBE
236.06103	$[C_{15}{}^{13}C_1H_{10}S + H]^+$	11.5	236.06095	0.32	# of heteroatoms

m/z	Composition	DBE	theor. *m/z*	ppm error	comment
236.06535	$C_{16}H_{12}S^{+\cdot}$	11.0	236.06542	-0.33	# of heteroatoms
236.14329	$[C_{17}H_{17}N + H]^+$	9.5	236.14338	-0.37	
237.06867	$C_{15}{}^{13}C_1H_{12}S^{+\cdot}$	11.0	237.06878	-0.44	# of heteroatoms
237.07313	$[C_{16}H_{12}S + H]^+$	10.5	237.07325	-0.50	# of heteroatoms
237.12724	$[C_{17}H_{16}O + H]^+$	9.5	237.12739	-0.65	# of heteroatoms
237.14678	$[C_{16}{}^{13}C_1H_{17}N + H]^+$	9.5	237.14673	0.22	isotopologue
237.15115	$C_{17}H_{19}N^{+\cdot}$	9.0	237.15120	-0.23	
237.15479	$C_{14}H_{23}NS^{+\cdot}$	4.0	237.15457	0.94	# of heteroatoms
237.16371	$[C_{18}H_{20} + H]^+$	8.5	237.16378	-0.30	ambiguous
238.07653	$[C_{15}{}^{13}C_1H_{12}S + H]^+$	10.5	238.07660	-0.29	# of heteroatoms
238.08094	$C_{16}H_{14}S^{+\cdot}$	10.0	238.08107	-0.55	# of heteroatoms
238.13525	$C_{17}H_{18}O^{+\cdot}$	9.0	238.13522	0.13	# of heteroatoms
239.08544	$[C_{19}H_{10} + H]^+$	14.5	239.08553	-0.34	too high DBE
248.05031	$C_{13}H_{12}O_3S^{+\cdot}$	8.0	248.05017	0.59	# of heteroatoms
250.08095	$C_{17}H_{14}S^{+\cdot}$	11.0	250.08107	-0.48	# of heteroatoms
250.99836	$[C_{15}H_6S_2 + H]^+$	12.5	250.99837	-0.04	# of heteroatoms
251.05250	$[C_{16}H_{10}OS + H]^+$	11.5	251.05251	-0.06	# of heteroatoms
251.06904	$C_{12}{}^{13}C_1H_{14}O_3S^{+\cdot}$	7.0	251.06917	-0.53	# of heteroatoms
251.07990	$C_{13}H_{17}NS_2^{+\cdot}$	6.0	251.07969	0.83	# of heteroatoms
251.08434	$C_{16}{}^{13}C_1H_{14}S^{+\cdot}$	11.0	251.08443	-0.34	# of heteroatoms
251.08890	$[C_{17}H_{14}S + H]^+$	10.5	251.08890	0.03	# of heteroatoms
251.14288	$[C_{18}H_{18}O + H]^+$	9.5	251.14304	-0.66	# of heteroatoms
251.16221	$[C_{17}{}^{13}C_1H_{19}N + H]^+$	9.5	251.16238	-0.69	isotopologue
251.16669	$C_{18}H_{21}N^{+\cdot}$	9.0	251.16685	-0.63	
251.17042	$C_{15}H_{25}NS^{+\cdot}$	4.0	251.17022	0.78	# of heteroatoms
251.17925	$[C_{19}H_{22} + H]^+$	8.5	251.17943	-0.70	ambiguous
261.05722	$C_{20}H_7N^{+\cdot}$	18.0	261.05730	-0.30	too high DBE
262.06491	$[C_{10}{}^{13}C_1H_{16}O_3S_2 + H]^+$	3.5	262.06472	0.75	# of heteroatoms
265.10114	$[C_{21}H_{12} + H]^+$	15.5	265.10118	-0.14	too high DBE
265.10438	$[C_{18}H_{16}S + H]^+$	10.5	265.10455	-0.65	# of heteroatoms
265.10502	$[C_{16}{}^{13}C_1H_{13}O_2N + H]^+$	11.5	265.10526	-0.92	# of heteroatoms
266.01499	$C_{22}H_2^{+\cdot}$	22.0	266.01510	-0.42	too high DBE
266.09632	$[C_{20}H_{11}N + H]^+$	15.5	266.09643	-0.40	# of carbon atoms
266.10901	$C_{21}H_{14}^{+\cdot}$	15.0	266.10900	0.05	too high DBE
266.19022	$[C_{19}H_{23}N + H]^+$	8.5	266.19033	-0.39	
